# A simple copper-catalyzed two-step one-pot synthesis of indolo[1,2-*a*]quinazoline

**DOI:** 10.3762/bjoc.10.254

**Published:** 2014-10-21

**Authors:** Chunpu Li, Lei Zhang, Shuangjie Shu, Hong Liu

**Affiliations:** 1 Department of Medicinal Chemistry, China Pharmaceutical University, 24 Tong Jia Xiang, Nanjing 210009, P. R. China; 2 CAS Key Laboratory of Receptor Research, Shanghai Institute of Materia Medica, Chinese Academy of Sciences, 555 Zuchongzhi Road, Shanghai 201203, P. R. China

**Keywords:** copper, one pot, synthetic methods

## Abstract

A convenient CuI/L-proline-catalyzed, two-step one-pot method has been developed for the preparation of indolo[1,2-*a*]quinazoline derivatives using a sequential Ullmann-type C–C and C–N coupling. This protocol provides an operationally simple and rapid strategy for preparing indolo[1,2-*a*]quinazoline derivatives and displays good functional group tolerance. All the starting materials are commercial available or can be easily prepared.

## Introduction

**Figure 1:**
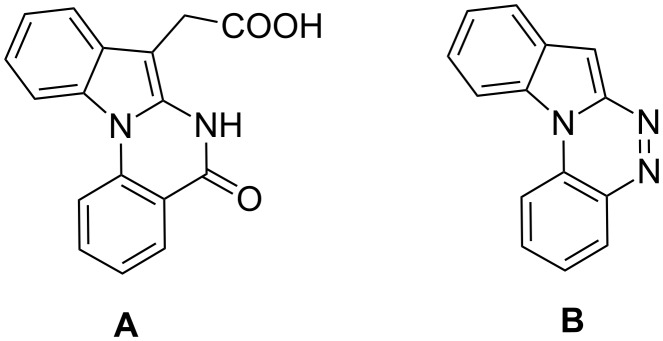
Representative examples of bioactive tetracyclic compounds containing the indole motif.

Indole motifs are important in natural products and pharmaceutical compounds [1–5]. In particular, tetracyclic compounds containing the indole substructure represent an important structural motif in a variety of bioactive compounds, such as antitumor agents A [6] and antifungal agents B [7] (Figure 1). Therefore, it is necessary to develop efficient and convenient methods to prepare nitrogen-containing tetracyclic compounds incorporating the bioactive indole motif in organic chemistry and medicinal chemistry.

**Scheme 1:**
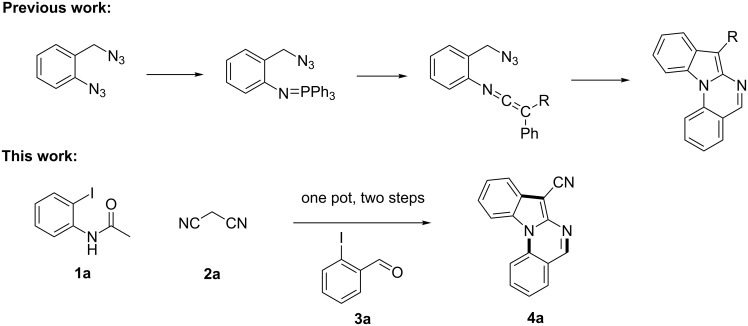
Synthetic route for indolo[1,2-*a*]quinazoline derivatives by a sequential Ullmann-type C–C and C–N coupling in one pot.

Over the past decades, copper catalysts have been proven highly powerful for various cross-coupling reactions, including Ullmann-type couplings of aryl halides with active methylene compounds such as ethyl acetoacetate, malononitrile, cyanoacetate and their equivalents [8–15]. Copper-catalyzed domino reactions have also been used in the synthesis of nitrogen-containing compounds [16–20]. Ma et al reported a convenient method for the synthesis of 2-(trifluoromethyl)indoles by introducing the trifluoroacetyl group to activate the CuI/L-proline-catalyzed system [21]. Zhao [22] and Kobayashi [23] reported the synthesis of 2-amino-1*H*-indole derivatives using the same kind of copper-catalyzed system. Meanwhile, the Ullmann condensation is a powerful method for C–N coupling [24–26], especially the *N*-arylation of nitrogen-containing heterocycles such as indoles [27–28]. Indolo[1,2-*a*]quinazoline is a kind of tetracyclic compounds containing the indole motif that has been constructed by intramolecular [3 + 2] cycloadditions of azido-ketenimines and azido-carbodiimides (Scheme 1) [29]. The available starting materials for the synthesis of these compounds, however, are limited. Very recently, Perumal [30] reported an efficient method for the synthesis of indolo[1,2-*a*]quinazoline through a Cu(I)-catalyzed intramolecular domino cyclization. Based on the previous work for the copper-catalyzed synthesis of 2-amino-1*H*-indole derivatives and copper-catalyzed *N*-arylation, we herein report a simple and efficient one-pot method to synthesize indolo[1,2-*a*]quinazolines by a sequential Ullmann-type C–C and C–N coupling. Compared to the previous methods [29–30], the advantages of our method are as following: (1) All the starting materials are commercially available or easily prepared. (2) Functionalized indolo[1,2-*a*]quinazoline derivatives can be synthesized, especially 7-cyano- or 7-sulfonyl-substituted indolo[1,2-*a*]quinazoline derivatives. (3) This protocol is performed as a two-step reaction in one pot.

## Results and Discussion

Substituted *N*-(2-iodophenyl)acetamides **1** were synthesized from substituted 2-iodoaniline by acetylation [31–32]. Substituted *o*-iodobenzaldehydes **3** were prepared from 2-iodobenzoic acid derivatives by reduction and PCC oxidation [33].

Initially, *N*-(2-iodophenyl)acetamide (**1a**), malononitrile (**2a**) and 2-iodobenzaldehyde (**3a**) were chosen as model substrates to optimize reaction conditions including the catalysts, bases and solvents under argon atmosphere. Based on the previous work [22], four copper catalysts were screened at 80 °C using L-proline as ligand, and K_2_CO_3_ as base in a mixed solvent of DMSO and H_2_O (volume ratio 1:1) (Table 1, entries 1–4). To our delight, the desired product **4a** was obtained in 36% yield using CuI as catalyst and 50% yield with Cu_2_O (Table 1, entries 1 and 4). Considering that the formation of imine occurs in the second step, the presence of water in this system may hinder the reaction. To account for this, DMSO was chosen as solvent, and a higher yield (72%) was obtained using CuI as the catalyst (Table 1, entry 6). The reactivity decreased slightly when K_2_CO_3_ was replaced with Cs_2_CO_3_ as the base (Table 1, entry 7). However, when a weaker base (K_3_PO_4_) or an organic base (DBU) was used, the conversions of starting materials were lower (Table 1, entries 8 and 9). Some other solvents were investigated, iPrOH resulted in only trace of product, while no product was detected with 1,4-dioxane and DMF led to low yield (18%) (Table 1, entries 10–12). Among the ligands screened, L-proline was more beneficial to the catalysis than L-hydroxyproline and picolinic acid (Table 1, entries 6, 13 and 14). When the reaction temperature was changed to 70 °C only traces of product were detected (Table 1, entry 15). Eventually, CuI, the inexpensive ligand L-proline and two equivalents of K_2_CO_3_ as the base in DMSO were identified as the most efficient system (Table 1, entry 6).

**Table 1:** Optimization of the reaction conditions.^a^

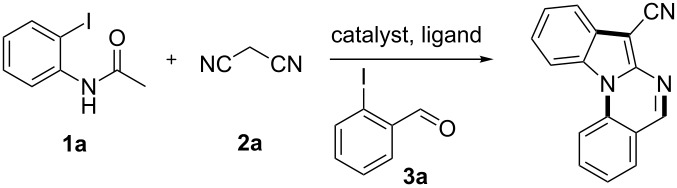					

Entry	Catalyst	Ligand^b^	Base	Solvent	Yield (%)^c^

1	CuI	A	K_2_CO_3_	DMSO/H_2_O^d^	36
2	CuBr	A	K_2_CO_3_	DMSO/H_2_O	21
3	Cu(OAc)_2_	A	K_2_CO_3_	DMSO/H_2_O	16
4	Cu_2_O	A	K_2_CO_3_	DMSO/H_2_O	50
5	Cu_2_O	A	K_2_CO_3_	DMSO	45
6	CuI	A	K_2_CO_3_	DMSO	72
7	CuI	A	Cs_2_CO_3_	DMSO	60
8	CuI	A	K_3_PO_4_	DMSO	N.D.
9	CuI	A	DBU	DMSO	N.D.
10	CuI	A	K_2_CO_3_	DMF	18
11	CuI	A	K_2_CO_3_	iPrOH	trace
12	CuI	A	K_2_CO_3_	1,4-dioxane	N.R.
13	CuI	B	K_2_CO_3_	DMSO	38
14	CuI	C	K_2_CO_3_	DMSO	31
15^e^	CuI	A	K_2_CO_3_	DMSO	trace

^a^Reaction conditions: **1a** (0.38 mmol ), **2a** (0.46 mmol, 1.2 equiv), catalyst (0.038 mmol, 0.1 equiv), ligand (0.076 mmol, 0.2 equiv), base (0.76 mmol, 2 equiv) in 0.77 mL of solvent under argon atmosphere at 80 °C for 12 h; then **3a** in 0.77 mL of solvent, another 12 h. ^b^A = L-proline, B = L-hydroxyproline, C = picolinic acid. ^c^Isolated yield. ^d^DMSO/H_2_O 1:1. ^e^Reaction temperature: 70 °C.

With the optimized conditions in hand, the scope of the copper-catalyzed reactions of substituted *N*-(2-iodophenyl)acetamides with malononitriles and substituted *o*-iodobenzaldehydes was investigated. As summarized in Table 2, the desired products **4a**–**4q** were obtained in moderate to good yields (34–72%) by treatment of various substituted *N*-(2-iodophenyl)acetamides **1a**–**1k** with active methylene compounds **2a**–**2c** and substituted *o*-iodobenzaldehydes **3a**–**3e**. For *N*-(2-iodophenyl)acetamide substrates, an electron-donating *p*-methyl group afforded a good isolated yield of the desired product (Table 2, entry 2). However, substrate **1c** with an electron-donating *p*-methoxy group was found to decrease the yield of the corresponding product (Table 2, entry 3). This result may be attributed to its low stability during the reaction. In comparison, electron-withdrawing *p*-trifluoromethyl and ester-substituted *N*-(2-iodophenyl)acetamides led to decreased yields of the desired compounds (Table 2, entries 4 and 5). Various halogens (F, Cl, Br) in *para-*position were well-tolerated on substrates **1** (Table 2, entries 6–8). Then, halogen-substituents (F, Cl) in meta position gave moderate yields (Table 2, entries 9 and 10). While a *m*-ester group on reactant **1k** resulted in a decreased yield (Table 2, entry 11). Other types of acetonitriles substituted with electron-withdrawing groups (–CO_2_Me, –SO_2_Me, –SO_2_Ph, and –PO(OEt)_2_) were also investigated. Unfortunately, –CO_2_Me and –PO(OEt)_2_ failed to afford the desired product under the same conditions, while –SO_2_Me and –SO_2_Ph produced moderate isolated yields of the target products (Table 2, entries 12 and 13). Furthermore, the catalytic system tolerated a variety of substituted *o*-iodobenzaldehydes in the reaction. For *o*-iodobenzaldehyde substrates, electron-donating methoxy groups decreased the yield (Table 2, entry 14). However, a methyl group at the *para*-position of iodine in reactant **3c** resulted in a good yield (Table 2, entry 15). Halogen-substituted (F, Cl) substrates **3** also provided the desired products with moderate yields (Table 2, entries 16 and 17).

**Table 2:** Synthesis of indolo[1,2-*a*]quinazolines **4**.^a^

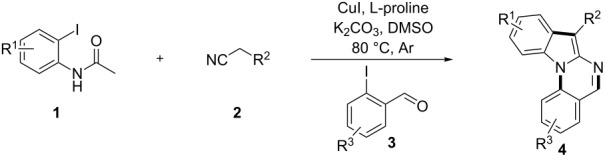					

Entry	**1**	**2**	**3**	Product	Yield (%)^b^

1	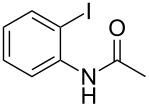 **1a**	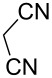 **2a**	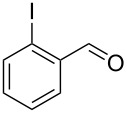 **3a**	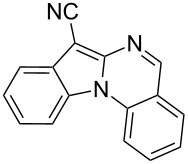 **4a**	72
2	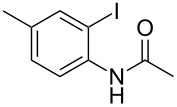 **1b**	**2a**	**3a**	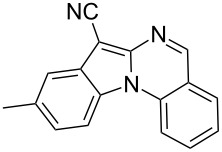 **4b**	71
3	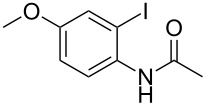 **1c**	**2a**	**3a**	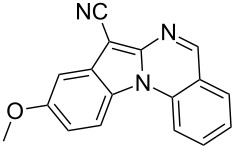 **4c**	45
4	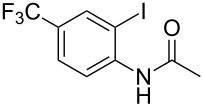 **1d**	**2a**	**3a**	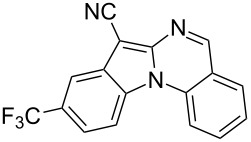 **4d**	49
5	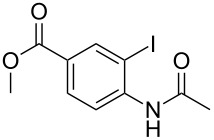 **1e**	**2a**	**3a**	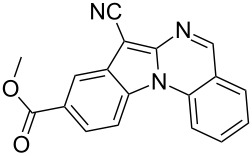 **4e**	51
6	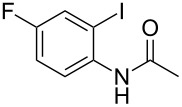 **1f**	**2a**	**3a**	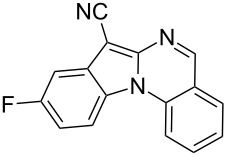 **4f**	63
7	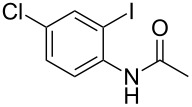 **1g**	**2a**	**3a**	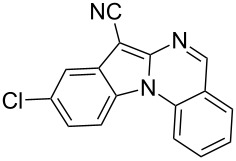 **4g**	49
8	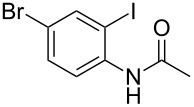 **1h**	**2a**	**3a**	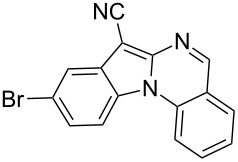 **4h**	56
9	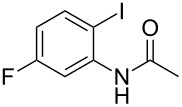 **1i**	**2a**	**3a**	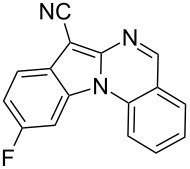 **4i**	51
10	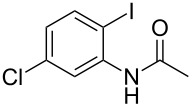 **1j**	**2a**	**3a**	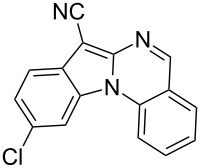 **4j**	54
11	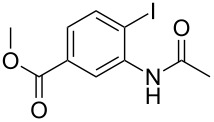 **1k**	**2a**	**3a**	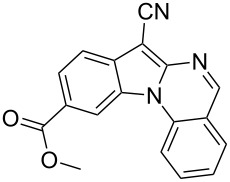 **4k**	37
12	**1a**	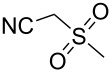 **2b**	**3a**	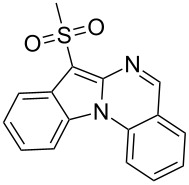 **4l**	52
13	**1a**	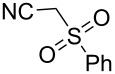 **2c**	**3a**	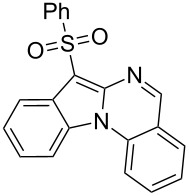 **4m**	53
14	**1a**	**2a**	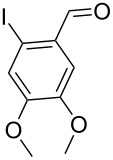 **3b**	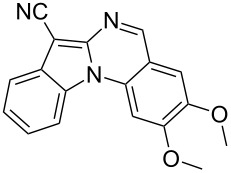 **4n**	32
15	**1a**	**2a**	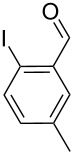 **3c**	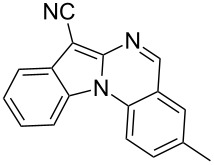 **4o**	64
16	**1a**	**2a**	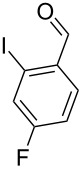 **3d**	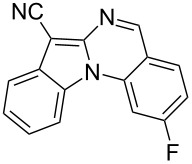 **4p**	54
17	**1a**	**2a**	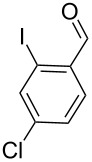 **3e**	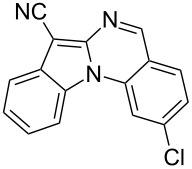 **4q**	55

^a^Reaction conditions: **1** (100 mg, 1 equiv), **2** (1.2 equiv), catalyst (0.1 equiv), ligand (0.2 equiv), base (2 equiv) in DMSO (0.5 M) under argon atmosphere at 80 °C for 12 h; then **3** in DMSO , another 12 h. ^b^Isolated yield.

## Conclusion

In conclusion, we have developed a simple and efficient Cu-catalyzed methodology for the synthesis of indolo[1,2-*a*]quinazoline derivatives. This approach produced nitrogen-containing tetracyclic compounds in moderate to good yields from simple starting materials. This method will provide an opportunity for the construction of diverse and useful nitrogen-containing tetracyclic compounds that incorporate the bioactive indole motif in organic chemistry and medicinal chemistry.

## Experimental

### General procedure for the synthesis of indolo[1,2-*a*]quinazolines **4a**–**4q**

A dry sealed tube was charged with a magnetic stirrer, substituted *N*-(2-iodophenyl)acetamide (100 mg for each example, 0.38 mmol), malononitrile or 2-sulfonylacetonitriles (0.46 mmol, 1.2 equiv), CuI (0.038 mmol, 0.1 equiv), L-proline (0.076 mmol, 0.2 equiv), and K_2_CO_3_ (0.76 mmol, 2 equiv) in 0.77 mL of DMSO. The tube was evacuated and backfilled with argon and the process was repeated three times. The mixture was stirred at 80 °C for 12 h under an argon atmosphere. After the starting material was consumed completely, 2-iodobenzaldehyde (0.4 mmol, 1.05 equiv) with 0.77 mL of DMSO was charged successively to the tube via syringe, and then the resulting mixture was stirred at 80 °C for another 12 h under an argon atmosphere. After the reaction was complete, the reaction mixture was cooled to room temperature and the reaction mixture was partitioned between ethyl acetate or dichloromethane and water. The organic layer was separated and the aqueous layer was extracted with ethyl acetate or dichloromethane for three times. The combined organic solution was washed with water, brine, dried over anhydrous Na_2_SO_4_, and concentrated under reduced pressure to give the crude product. Purification by chromatography on silica gel using petroleum ether/ethyl acetate or dichloromethane/ethyl acetate as eluent provided the desired product.

## Supporting Information

File 1 General information, experimental details, characterization data and copies of ^1^H and ^13^C NMR spectra.
