# Supplementary material for: A simple copper-catalyzed two-step one-pot synthesis of indolo[1,2-a]quinazoline
Source: Beilstein J Org Chem. 2014 Oct 21;10:2441–7. doi: 10.3762/bjoc.10.254 (PMC4222401; doi:10.3762/bjoc.10.254)
Supplement: File 1 — General information, experimental details, characterization data and copies of 1H and 13C NMR spectra. [file Beilstein_J_Org_Chem-10-2441-s001.pdf]

Supporting information
for
A simple copper-catalyzed two-step one-pot synthesis of indolo[1,2-*a*]quinazoline

Chunpu Li[1,2], Lei Zhang [2], Shuangjie Shu[2] and Hong Liu*[1,2]

Address: [1]Department of Medicinal Chemistry, China Pharmaceutical University, 24 Tong Jia Xiang, Nanjing 210009, P. R. China and [2]CAS Key Laboratory of Receptor Research, Shanghai Institute of Materia Medica, Chinese Academy of Sciences, 555 Zuchongzhi Road, Shanghai 201203, P. R. China

Email: Hong Liu* - hliu@mail.shcnc.ac.cn

*Corresponding author

General information, experimental details, characterization data and copies of ^{1}H and ^{13}C NMR spectra

Table of Contents

General Information S2

Preparation and characterization data of compounds **4a**–**4q** S2–S7

References S7

^{1}H and ^{13}C NMR spectra S8–S24

General Information

Unless otherwise stated, all reagents used were commercially purchased without further purification. DMSO is commercially available without further evaporation. Substrates **1a-1k** and **3a-3e** were prepared according to the known literature procedure [1-7]. Analytical thin layer chromatography (TLC) was HSGF 254 (0.15-0.2 mm thickness). Compound spots were visualized by UV light (254 nm). Column chromatography was performed on silica gel FCP 200-300. NMR spectra were run on 400 or 500 MHz instrument. Chemical shifts were reported in parts per million (ppm, δ) downfield from tetramethylsilane. Proton coupling patterns are described as singlet (s), doublet (d), triplet (t), quartet (q), multiplet (m), and broad (br). High-resolution mass spectra (HRMS) was measured on Micromass Ultra Q-TOF spectrometer.

Preparation and characterization data of the compounds 4a-4q

Procedure for the preparation of indolo[1,2-*a*]quinazoline (4a).

A dry sealed tube was charged with a magnetic stirrer, substituted *N*-(2-iodophenyl)acetamide (100 mg for each example, 0.38 mmol), malononitrile or 2-sulfonylacetonitriles (0.46 mmol, 1.2 equiv), CuI (0.038 mmol, 0.1equiv), L-proline (0.076 mmol, 0.2 equiv), and K_2CO_3 (0.76 mmol, 2 equiv) in 0.77 mL of DMSO. The tube was evacuated and backfilled with argon and the process was repeated three times. The mixture was stirred at 80 °C for 12 h under a argon atmosphere. After the starting material was consumed completely, 2-iodobenzaldehyde (0.4 mmol, 1.05 equiv) with 0.77 mL of DMSO was charged successively to the tube via syringe, and then the resulting mixture was stirred at 80 °C for another 12 h under an argon atmosphere. After the reaction was complete, the reaction mixture was cooled to room temperature, the reaction mixture was partitioned between ethyl acetate or dichloromethane and water. Organic layer was separated, and aqueous layer was extracted with ethyl acetate or dichloromethane for three times, the combined organic solution was washed with water, brine, dried over anhydrous Na_2SO_4, and concentrated under vacuum to give crude product. The crude product was purified by chromatography on silica gel (Petroleum ether/EtOAc = 4:1 as eluent) to give **4a**. Yellow solid (67mg, 72%), mp 219-220 °C; ^{1}H NMR (500 MHz, [D6]DMSO) δ 9.12 (s, 1H), 8.70 (d, J = 8.0 Hz, 1H), 8.62 (s, 1H), 8.17 (d, J = 7.5 Hz, 1H),

8.01 (t, J = 7.4 Hz, 1H), 7.83 (s, 1H), 7.64 (t, J = 7.5 Hz, 1H), 7.54 (d, J = 3.0 Hz, 2H); ^{13}C NMR (126 MHz, [D6]DMSO) δ 156.56, 146.69, 136.15, 135.59, 130.44, 129.07, 127.41, 125.33, 124.54, 124.50, 119.05, 118.77, 115.66, 115.63, 115.14, 78.23; MS (ESI, *m*/*z*) 244 $[M+H]^+$; HRMS (ESI): calcd.for $C_{16}H_9N_3Na$ $[M+Na]^+$ 266.0694, found 266.0696.

Compounds 4b-4p were prepared following the similar procedure carried out for 4a.

9-Methylindolo[1,2-*a*]quinazoline-7-carbonitrile (4b): Yellow solid (66mg, 71%), mp 230-232 °C; ^{1}H NMR (400 MHz, CF_3CO_2D) δ 9.54 (s, 1H), 8.86 (d, J = 8.4 Hz, 1H), 8.50-8.44 (m, 3H), 7.90–7.87 (m, 2H), 7.68 (d, J = 8.8 Hz, 1H), 2.61 (s, 3H); ^{13}C NMR (101 MHz, CF_3CO_2D) δ 154.49, 146.19, 141.95, 141.81, 137.39, 136.99, 131.97, 131.18, 130.24, 130.05, 122.05, 118.50, 118.35, 116.81, 77.92, 22.07; MS (ESI, *m*/*z*) 258 $[M+H]^+$; HRMS (ESI): calcd.for $C_{17}H_{11}N_3Na$ $[M+Na]^+$ 280.0851, found 280.0843.

9-Methoxyindolo[1,2-*a*]quinazoline-7-carbonitrile (4c): Yellow solid (42 mg, 45%), mp 228-230 °C; ^{1}H NMR (400 MHz, CF_3CO_2D) δ 9.42 (s, 1H), 8.74 (d, J = 8.4, 1H), 8.47 – 8.29 (m, 3H), 7.81 (t, J = 7.2, 1H), 7.49 – 7.34 (m, 2H), 3.98 (s, 3H); ^{13}C NMR (101 MHz, CF_3CO_2D) δ 161.05, 154.11, 145.81, 141.41, 138.16, 137.08, 131.91, 130.14, 127.99, 120.72, 118.73, 118.63, 118.27, 103.64, 78.19, 57.87; MS (ESI, *m*/*z*) 274 $[M+H]^+$; HRMS (ESI): calcd.for $C_{17}H_{11}N_3ONa$ $[M+Na]^+$ 296.0800, found 296.0805.

9-(Trifluoromethyl)indolo[1,2-*a*]quinazoline-7-carbonitrile (4d): Yellow solid (46 mg, 49%), mp 271-273 °C; ^{1}H NMR (400 MHz, CF_3CO_2D) δ 9.48-9.41 (m, 1H), 8.78-8.71 (m, 1H), 8.64-8.57 (m, 1H), 8.41 – 8.29 (m, 2H), 8.27-8.21 (m, 1H), 7.95-7.88 (m, 1H), 7.86-7.78 (m, 1H); ^{13}C NMR (101 MHz, CF_3CO_2D) δ 157.99, 144.20, 142.31, 140.93, 136.55, 133.80, (132.23, 131.88-CF3), 130.13, 129.59, 127.17, 125.57, 124.47, 120.09, 119.61, 118.18, 117.94, 79.67; MS (EI, *m*/*z*) 311 $[M]^+$; HRMS (EI): calcd.for $C_{17}H_8N_3F_3$ $[M]^+$ 311.0662, found 311.0666.

Methyl 7-cyanoindolo[1,2-*a*]quinazoline-9-carboxylate (4e): Yellow solid (48 mg, 51%), mp 298-300 °C; ^{1}H NMR (400 MHz, CF_3CO_2D) δ 9.32 (s, 1H), 8.65 (d, J = 8.4 Hz, 1H), 8.55

(s, 1H), 8.48 (d, *J* = 8.8 Hz, 1H), 8.30-8.21 (m, 3H), 7.74 (t, *J* = 6.8 Hz, 1H), 3.99 (s, 3H); ^{13}C NMR (101 MHz, CF_3CO_2D) δ 171.79 , 158.42, 144.07, 143.05, 140.82, 136.54, 135.18, 130.30, 129.75, 129.69, 129.62, 125.10, 119.89, 118.43, 117.77, 113.33, 80.00, 55.55; MS (EI, *m*/*z*) 301 $[M]^+$; HRMS (EI): calcd.for $C_{18}H_{11}N_3O_2$ $[M]^+$301.0867, found 301.0859.

9-Fluoroindolo[1,2-*a*]quinazoline-7-carbonitrile (4f): Yellow solid (59 mg, 63%), mp 281-282 °C; ^{1}H NMR (400 MHz, CF_3CO_2D) δ 9.47 (s, 1H), 8.75 (d, *J* = 8.8 Hz, 1H), 8.50 (dd, *J* = 9.4, 3.6 Hz, 1H), 8.42-8.35 (m, 2H), 7.83 (t, *J* = 7.6 Hz, 1H), 7.61 (dd, *J* = 7.6, 2.2 Hz, 1H), 7.46 (td, *J* = 9.2, 2.4 Hz, 1H); ^{13}C NMR (101 MHz, CF_3CO_2D) δ 163.91 (d, *J* = 251.5 Hz), 155.95, 145.48, 141.33, 139.77, 137.04, 131.50 (d, *J* = 11.1 Hz), 130.15, 129.25, 119.11 (d, *J* = 10.1 Hz), 118.90, 118.60 (d, *J* = 26.3 Hz), 118.06, 107.91 (d, *J* = 26.3 Hz), 78.62; MS (EI, *m*/*z*) 261 $[M]^+$; HRMS (EI): calcd.for $C_{16}H_8FN_3$ $[M]^+$ 261.0712, found 261.0707.

9-Chloroindolo[1,2-*a*]quinazoline-7-carbonitrile (4g): Yellow solid (46 mg, 49%), mp 276-278 °C; ^{1}H NMR (400 MHz, CF_3CO_2D) δ 9.36 (s, 1H), 8.60 (d, *J* = 8.9 Hz, 1H), 8.37 – 8.24 (m, 3H), 7.77 (t, *J* = 7.6 Hz, 1H), 7.69 (d, *J* = 1.8 Hz, 1H), 7.52 (dd, *J* = 9.2, 1.9 Hz, 1H); ^{13}C NMR (101 MHz, CF_3CO_2D) δ 156.83, 144.73, 140.92, 140.78, 136.73, 136.20, 130.87, 130.78, 130.13, 129.90, 121.72, 119.26, 118.26, 118.11, 78.09; MS (EI, *m*/*z*) 277 $[M]^+$; HRMS (EI): calcd.for $C_{16}H_8ClN_3$ $[M]^+$ 277.0457, found 277.0432.

9-Bromoindolo[1,2-*a*]quinazoline-7-carbonitrile (4h): Yellow solid (53 mg, 56%), mp >300 °C; ^{1}H NMR (400 MHz, CF_3CO_2D) δ 9.40 (s, 1H), 8.62 (d, *J* = 8.9 Hz, 1H), 8.34 (m, 2H), 8.25 (d, *J* = 9.2 Hz, 1H), 7.87 (s, 1H), 7.81 (t, *J* = 7.7 Hz, 1H), 7.68 (d, *J* = 9.2 Hz, 1H); ^{13}C NMR (101 MHz, CF_3CO_2D) δ 156.96, 144.57, 140.83, 136.66, 132.52, 131.10, 131.06, 130.08, 124.88, 123.32, 119.29, 118.30, 77.90; MS (EI, *m*/*z*) 321, 323 $[M]^+$; HRMS (EI): calcd.for $C_{16}H_8BrN_3$ $[M]^+$ 320.9891, found 320.9896.

10-Fluoroindolo[1,2-*a*]quinazoline-7-carbonitrile (4i): Yellow solid (48 mg, 51%), mp 260-261 °C; ^{1}H NMR (500 MHz, [D6]DMSO) δ 9.12 (s, 1H), 8.70 (d, *J* = 8.5 Hz, 1H), 8.55 (dd, *J* = 11.0, 2.0 Hz, 1H), 8.19 (dd, *J* = 7.7, 1.3 Hz, 1H), 8.05 – 7.95 (m, 1H), 7.88 (dd, *J* =

8.8, 5.5 Hz, 1H), 7.67 (t, *J* = 7.5 Hz, 1H), 7.45 (td, *J* = 9.0, 2.0 Hz, 1H); ^{13}C NMR (126 MHz, [D6]DMSO) *δ* 159.74 (d, *J* = 239.4 Hz), 156.54, 147.29, 135.78, 135.60, 130.44, 128.56 (d, *J* = 11.3 Hz), 125.59, 123.85, 120.42(d, *J* = 10.1 Hz), 118.73, 115.74, 114.80, 113.32 (d, *J* = 25.2 Hz), 102.66 (d, *J* = 29.0 Hz), 78.26; MS (EI, *m*/*z*) 261 $[M]^+$; HRMS (EI): calcd.for $C_{16}H_8FN_3$ $[M]^+$ 261.0684, found 261.0693.

10-Chloroindolo[1,2-*a*]quinazoline-7-carbonitrile (4j): Yellow solid (51 mg, 54%), mp 265-267 °C; ^{1}H NMR (500 MHz, [D6]DMSO) *δ* 9.21 (s, 1H), 8.88 – 8.72 (m, 2H), 8.25 (d, *J* = 7.8 Hz, 1H), 8.06 (t, *J* = 7.8 Hz, 1H), 7.91 (d, *J* = 8.5 Hz, 1H), 7.71 (t, *J* = 7.5 Hz, 1H), 7.61 (d, *J* = 8.5 Hz, 1H); ^{13}C NMR (126 MHz, [D6]DMSO) *δ* 157.15, 147.32, 135.90, 130.57, 130.37, 129.24, 129.05, 126.12, 125.76, 125.08, 120.43, 118.90, 116.06, 115.34, 114.71, 78.41; MS (EI, *m*/*z*) 277 $[M]^+$; HRMS (EI): calcd.for $C_{16}H_8ClN_3$ $[M]^+$ 277.0443; found 277.0425.

Methyl 7-cyanoindolo[1,2-*a*]quinazoline-10-carboxylate (4k): Yellow solid (35 mg, 37%), mp 292-294 °C; ^{1}H NMR (400 MHz, CF_3CO_2D) *δ* 9.40 (s, 1H), 9.17 (s, 1H), 8.78 (d, *J* = 8.8 Hz, 1H), 8.37 – 8.22 (m, 3H), 7.95 (d, *J* = 8.4 Hz, 1H), 7.79 (t, *J* = 7.6 Hz, 1H), 4.06 (s, 3H); ^{13}C NMR (101 MHz, CF_3CO_2D) *δ* 172.10, 158.66, 144.24, 144.09, 140.76, 136.28, 134.18, 132.03, 130.17, 129.42, 129.27, 122.61, 119.88, 119.82, 118.46, 79.39, 55.70; MS (EI, *m*/*z*) 301 $[M]^+$; HRMS (EI): calcd.for $C_{18}H_{11}N_3O_2$ $[M]^+$ 301.0831; found 301.0841.

7-(Methylsulfonyl)indolo[1,2-*a*]quinazoline (4l): Yellow solid (59 mg, 52%), mp 242-244 °C; ^{1}H NMR (400 MHz, CF_3CO_2D) *δ* 9.56 (s, 1H), 8.91 (d, *J* = 8.8 Hz, 1H), 8.59 (d, *J* = 8.4 Hz, 1H), 8.55 – 8.43 (m, 2H), 8.16 (d, *J* = 7.6 Hz, 1H), 7.90 (t, *J* = 7.6 Hz, 1H), 7.87 – 7.74 (m, 2H), 3.47 (s, 3H); ^{13}C NMR (101 MHz, CF_3CO_2D) *δ* 155.60, 147.24, 142.13, 138.07, 133.37, 133.20, 130.63, 130.48, 130.26, 126.97, 121.86, 119.06, 118.45, 117.66, 102.75, 46.79; MS (EI, *m*/*z*) 296 $[M]^+$; HRMS (EI): calcd.for $C_{16}H_{12}N_2O_2S$ $[M]^+$ 296.0628, found 296.0624.

7-(Phenylsulfonyl)indolo[1,2-*a*]quinazoline (4m): Yellow solid (73 mg, 53%), mp 294-296 °C; ^{1}H NMR (400 MHz, CF_3CO_2D) δ 9.63 (s, 1H), 8.85 (d, J = 8.6 Hz, 1H), 8.54-8.44 (m, 3H), 8.07-7.95 (m, 3H), 7.88 (t, J = 7.6 Hz, 1H), 7.78-7.55 (m, 3H), 7.54 – 7.38 (m, 2H); ^{13}C NMR (101 MHz, CF_3CO_2D) δ 155.60, 147.20, 142.20, 141.31, 138.05, 137.68, 133.43, 132.99, 132.13, 130.55, 130.11, 128.91, 126.71, 122.13, 118.99, 118.45, 117.43, 103.92; MS (EI, *m*/*z*) 358 $[M]^+$; HRMS (EI): calcd.for $C_{21}H_{14}N_2O_2S$ $[M]^+$ 358.0742, found 358.0759.

2,3-Dimethoxyindolo[1,2-*a*]quinazoline-7-carbonitrile (4n): Yellow solid (38 mg, 32%), mp 264-266 °C; ^{1}H NMR (400 MHz, CF_3CO_2D) δ 9.18 (s, 1H), 8.42 – 8.35 (m, 1H), 8.19 (s, 1H), 8.00 – 7.94 (m, 1H), 7.72-7.65 (m, 3H), 4.30 (s, 3H), 4.03 (s, 3H); ^{13}C NMR (101 MHz, CF_3CO_2D) δ 166.86, 151.61, 150.01, 141.36, 137.72, 132.13, 130.29, 130.15, 129.26, 122.67, 116.97, 114.13, 113.14, 100.57, 75.88, 59.62, 58.38; MS (EI, *m*/*z*) 303 $[M]^+$; HRMS (EI): calcd.for $C_{18}H_{13}N_3O_2$ $[M]^+$ 303.1010, found 303.1009.

3-Methylindolo[1,2-*a*]quinazoline-7-carbonitrile (4o): Yellow solid (63 mg, 64%), mp 268-270 °C; ^{1}H NMR (400 MHz, CF_3CO_2D) δ 9.33 (s, 1H), 8.65 (d, J = 8.8 Hz, 1H), 8.40 (d, J = 8.4 Hz, 1H), 8.20 (d, J = 8.8 Hz, 1H), 8.09 (s, 1H), 7.90 (d, J = 8.0 Hz, 1H), 7.74 – 7.52 (m, 2H), 2.50 (s, 3H); ^{13}C NMR (101 MHz, CF_3CO_2D) δ 155.01, 148.02, 142.40, 140.28, 137.54, 136.58, 132.89, 130.04, 129.99, 129.94, 122.77, 121.09, 118.81, 118.56, 117.37, 78.01, 21.42; MS (EI, *m*/*z*) 257 $[M]^+$; HRMS (EI): calcd.for $C_{17}H_{11}N_3$ $[M]^+$ 257.0949, found 257.0951.

2-Fluoroindolo[1,2-*a*]quinazoline-7-carbonitrile (4p): Yellow solid (54 mg, 54%), mp 287-289 °C; ^{1}H NMR (400 MHz, CF_3CO_2D) δ 9.35 (s, 1H), 8.40-8.29 (m, 3H), 7.91 (d, J = 7.6 Hz, 1H), 7.72 – 7.58 (m, 2H), 7.44 (t, J = 8.0 Hz, 1H); ^{13}C NMR (101 MHz, CF_3CO_2D) δ 174.54 (d, J = 272.7 Hz), 154.74, 144.44 (d, J = 14.1 Hz), 140.72 (d, J = 13.1 Hz), 138.41, 132.71, 130.09, 130.00, 129.92, 122.82, 119.30 (d, J = 24.2 Hz), 116.95, 115.82, 106.43 (d, J = 29.3 Hz), 79.10; MS (EI, *m*/*z*) 261 $[M]^+$; HRMS (EI): calcd.for $C_{16}H_8FN_3$ $[M]^+$ 261.0677, found 261.0690.

2-Chloroindolo[1,2-*a*]quinazoline-7-carbonitrile (4q): Yellow solid (58 mg, 55%), mp 283-284 °C; ^{1}H NMR (400 MHz, CF_3CO_2D) δ 9.29 (s, 1H), 8.61 (s, 1H), 8.29 (d, J = 8.4 Hz, 1H), 8.19 (d, J = 8.4 Hz, 1H), 7.86 (d, J = 7.6 Hz, 1H), 7.74 – 7.54 (m, 3H); ^{13}C NMR (101 MHz, CF_3CO_2D) δ 155.17, 153.12, 141.70, 139.54, 137.46, 132.53, 130.72, 129.86, 129.68, 122.67, 118.63, 117.39, 116.90, 79.24; MS (EI, *m*/*z*) 277 $[M]^+$; HRMS (EI): calcd.for $C_{16}H_8ClN_3$ $[M]^+$ 277.0441, found 277.0424.

References

1. Hoque, M. M.; Halim, M. A.; Rahman, M. M.; Hossain, M. I.; Khan, M. W. *J. Mol. Struct.* **2013**, *1054-1055*, 367-374.
2. Bruch, A.; Frohlich, R.; Grimme, S.; Studer, A.; Curran, D. P. *J. Am. Chem. Soc.* **2011**, *133*, 16270-16276.
3. Kotha, S.; Shah, V. R. *Eur. J. Org. Chem.* **2008**, *6*, 1054-1064.
4. Shimada, T.; Nakamura, I.; Yamamoto, Y. *J. Am. Chem. Soc.* **2004**, *126*, 10546-10547.
5. Gimbert, C.; Vallribera, A. *Org. Lett.* **2009**, *11*, 269-271.
6. Zheng, N.; Buchwald, S. L. *Org. Lett.* **2007**, *9*, 4749-4751.
7. Zhou, P. X.; Luo, J. Y.; Zhao, L. B.; Ye, Y. Y.; Liang, Y. M. *Chem. Commun.* **2013**, *49*, 3254-3256.

^{1}H and ^{13}C NMR spectra

Indolo[1,2-*a*]quinazoline-7-carbonitrile (4a)

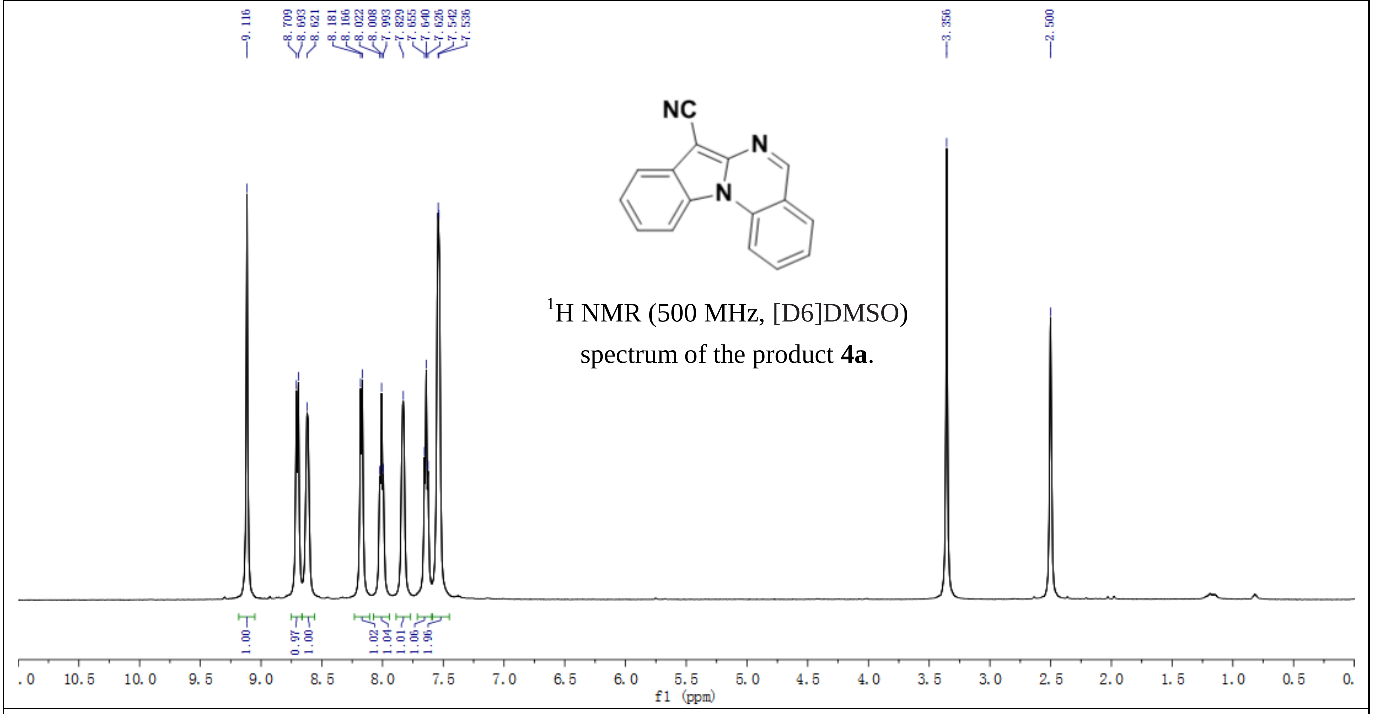

^{1}H NMR (500 MHz, [D6]DMSO) spectrum of the product **4a**.

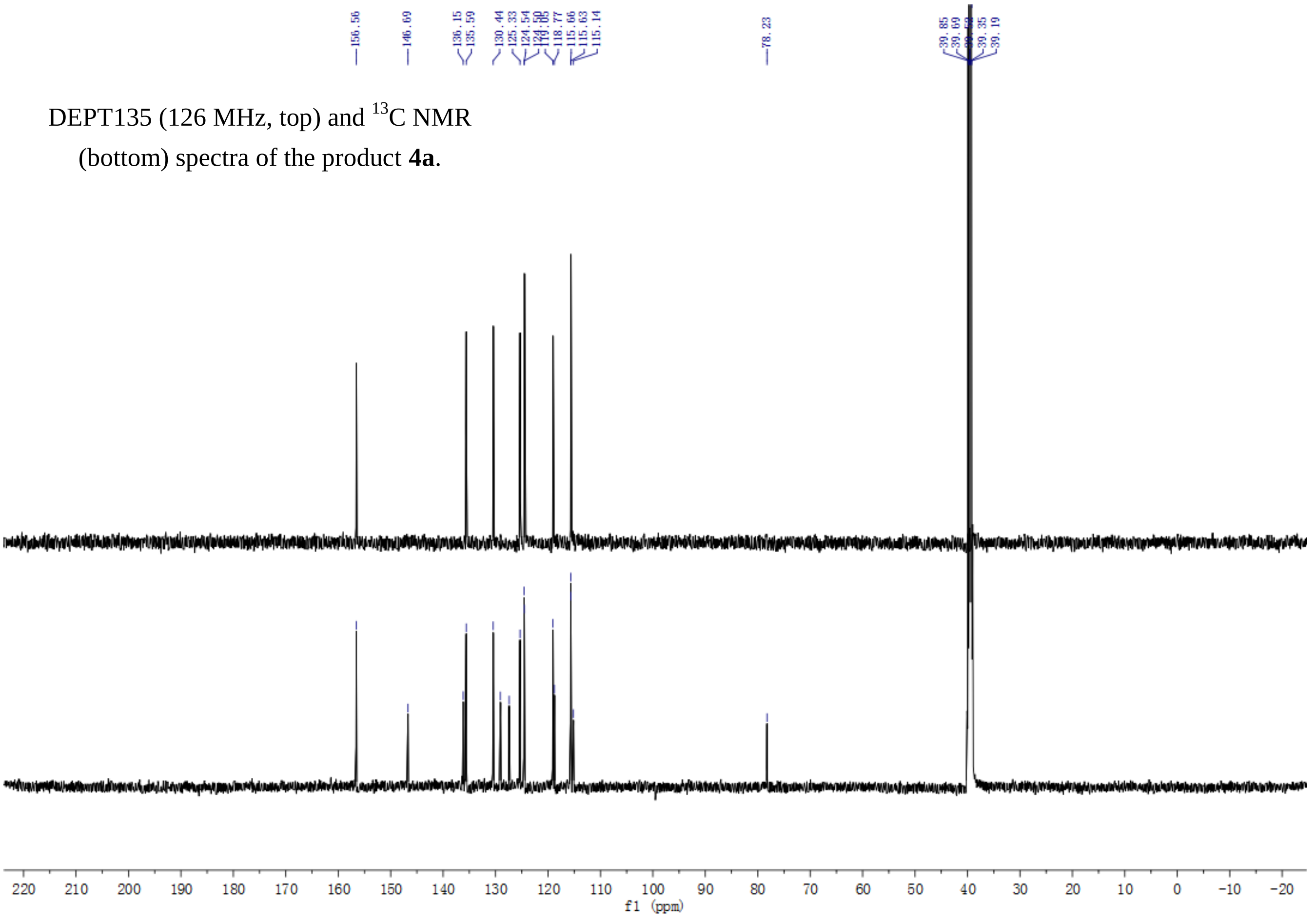

DEPT135 (126 MHz, top) and ^{13}C NMR (bottom) spectra of the product **4a**.

9-Methylindolo[1,2-*a*]quinazoline-7-carbonitrile (4b)

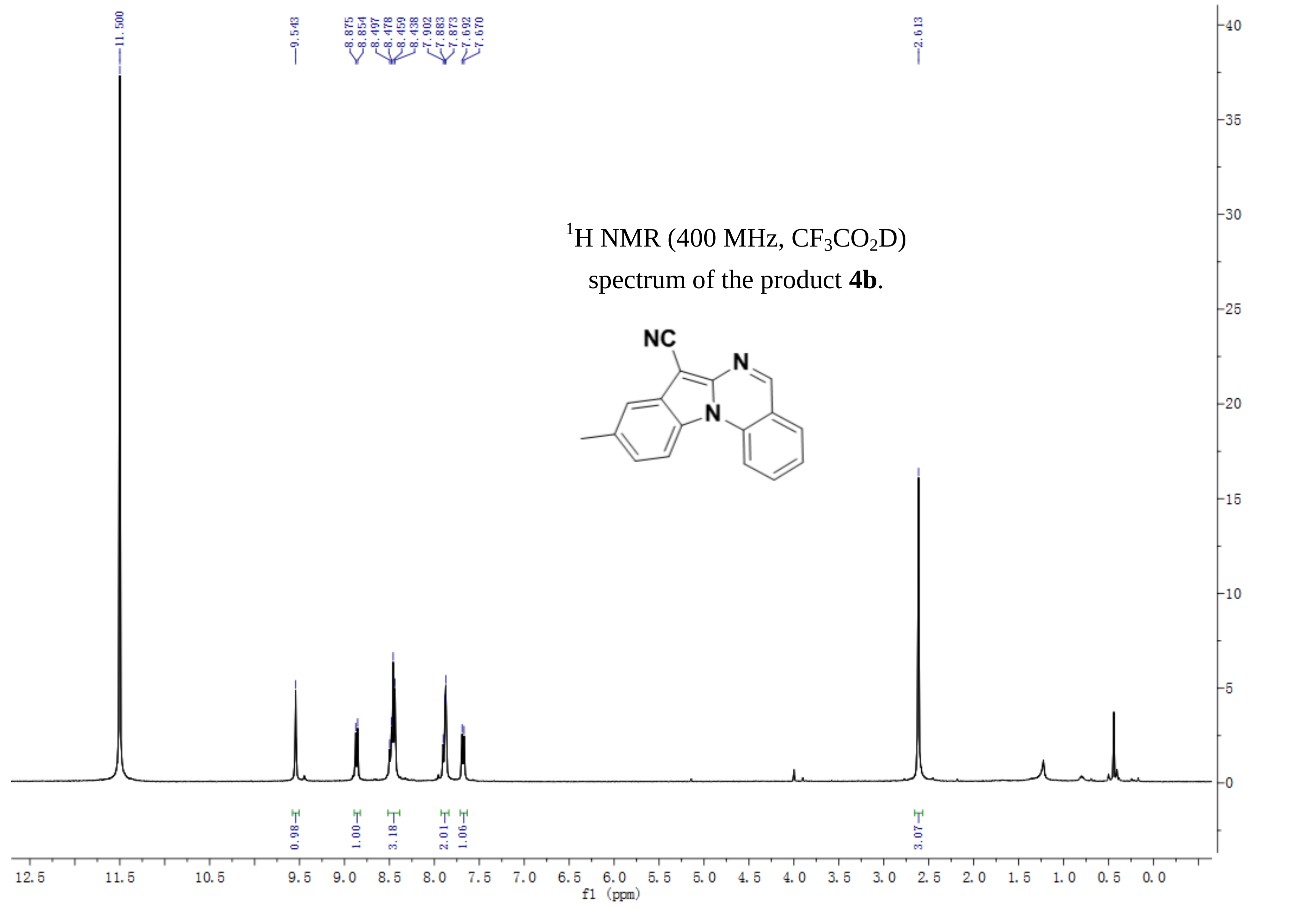

^{1}H NMR (400 MHz, CF_3CO_2D) spectrum of the product **4b**.

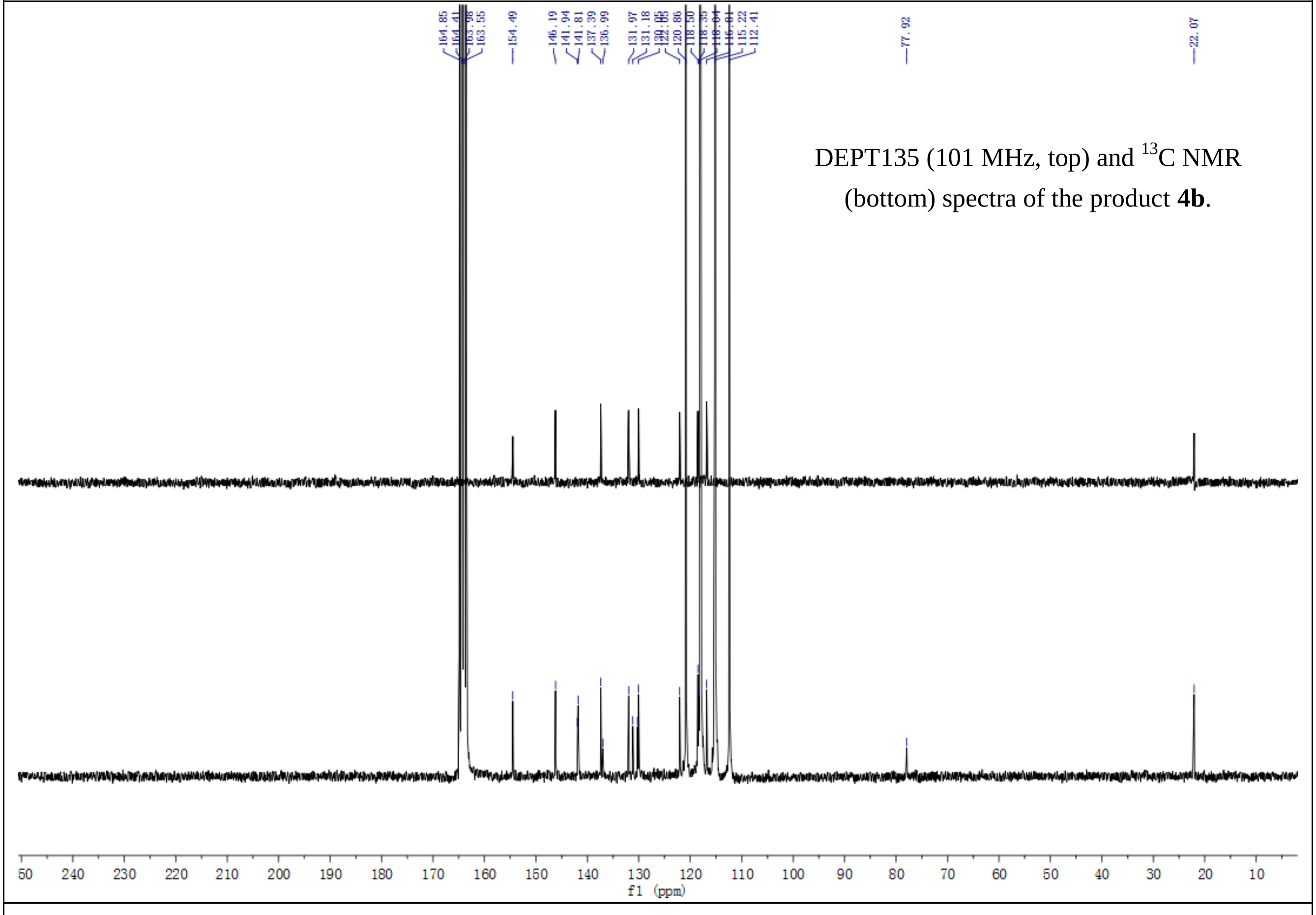

DEPT135 (101 MHz, top) and ^{13}C NMR (bottom) spectra of the product **4b**.

9-Methoxyindolo[1,2-*a*]quinazoline-7-carbonitrile (4c)

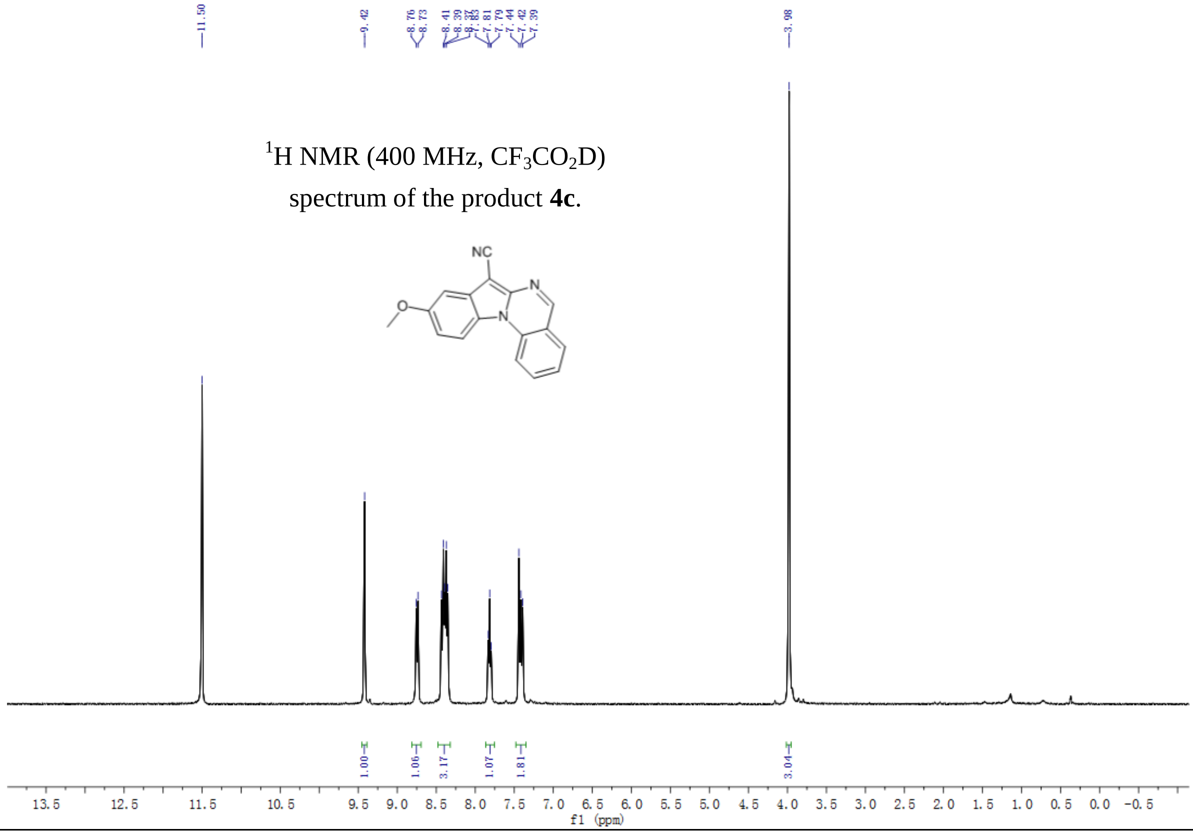

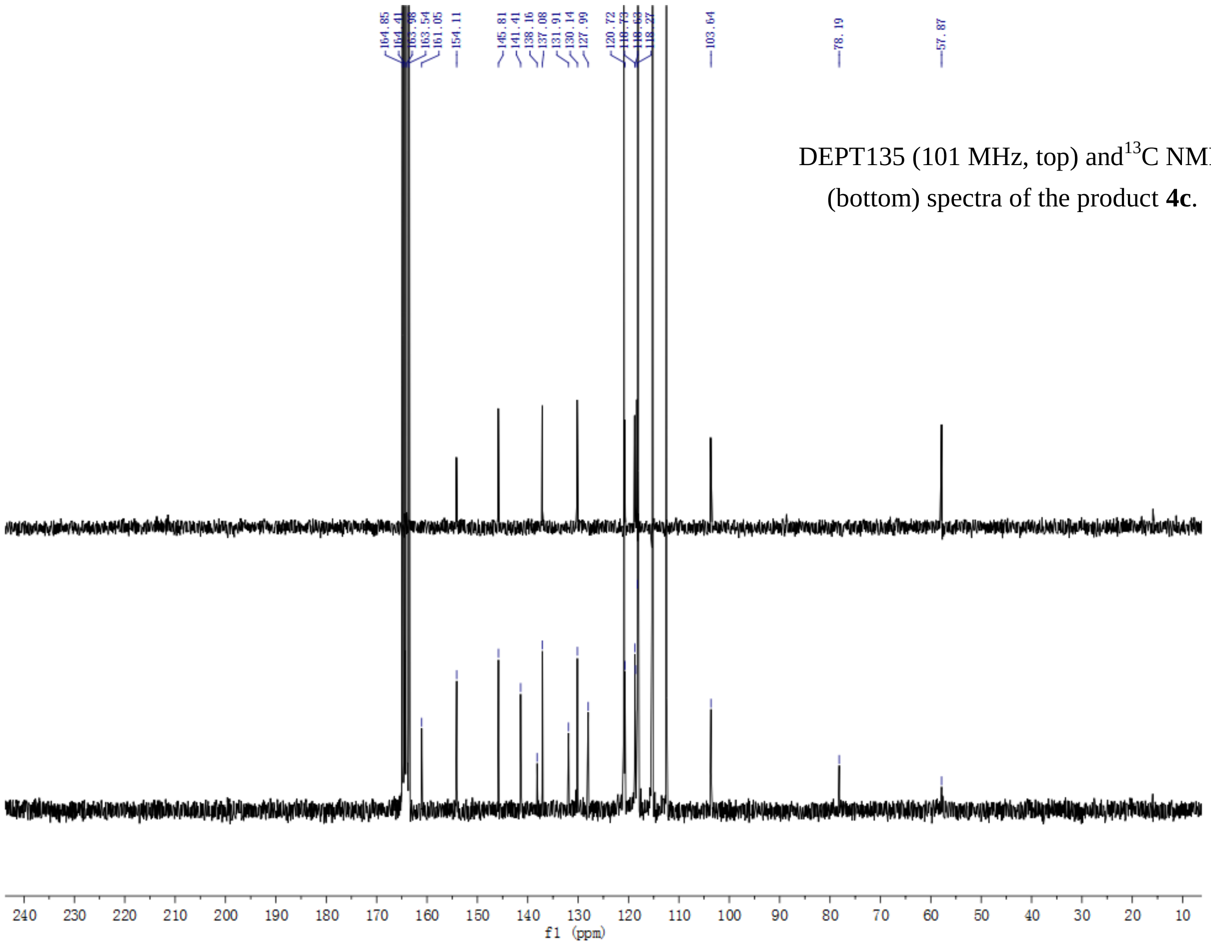

9-(Trifluoromethyl)indolo[1,2-*a*]quinazoline-7-carbonitrile (4d)

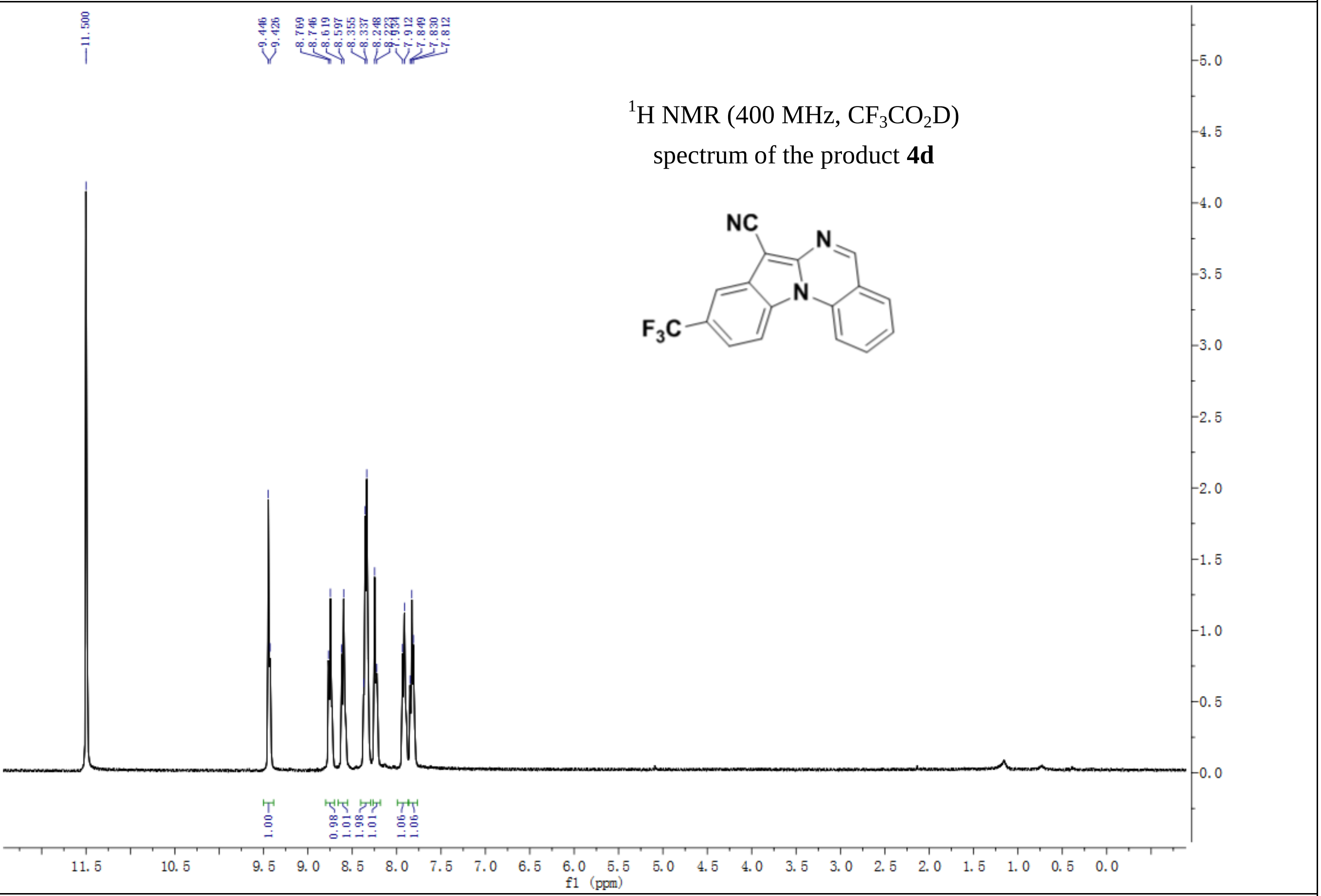

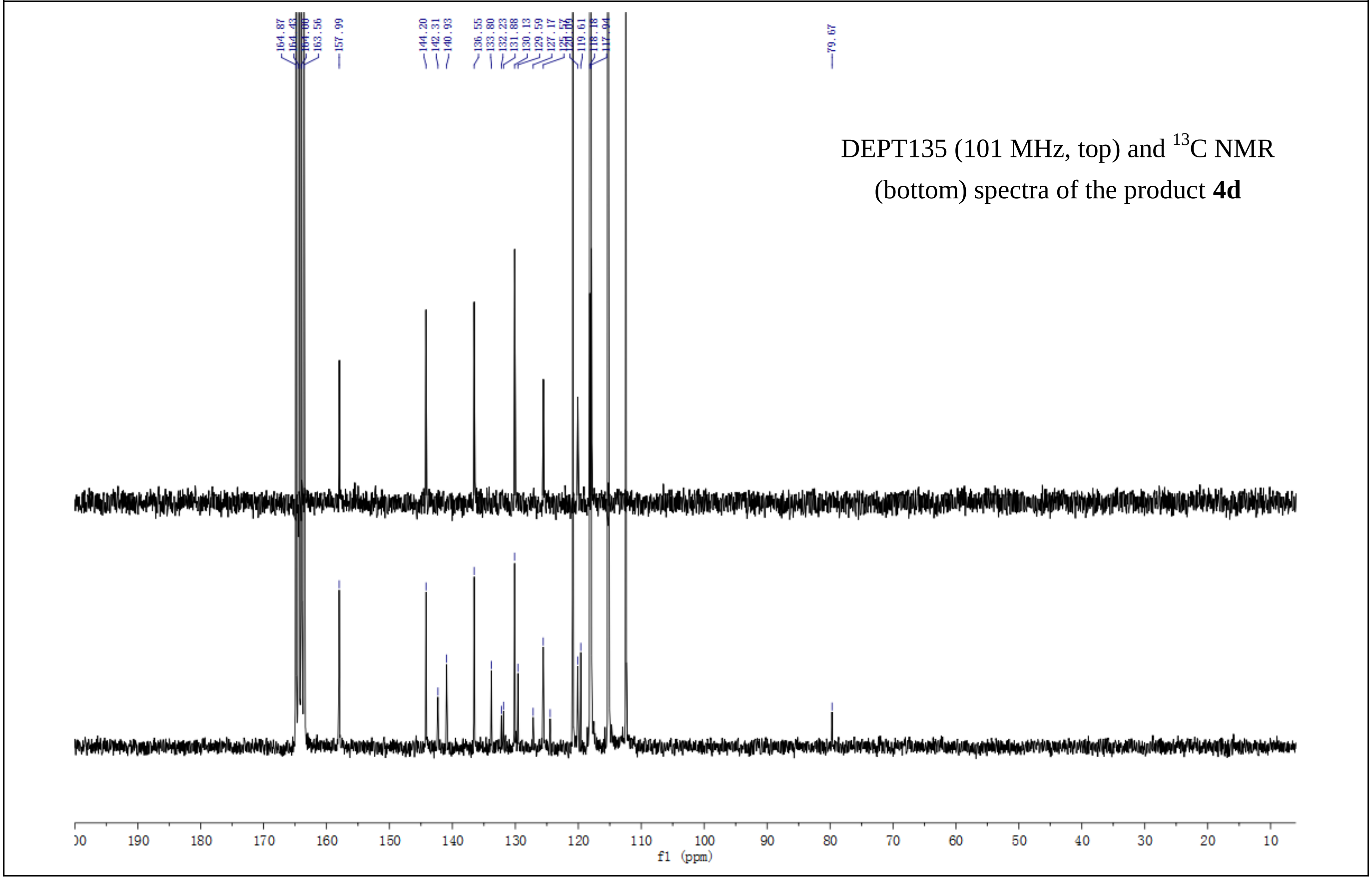

Methyl 7-cyanoindolo[1,2-*a*]quinazoline-9-carboxylate (4e)

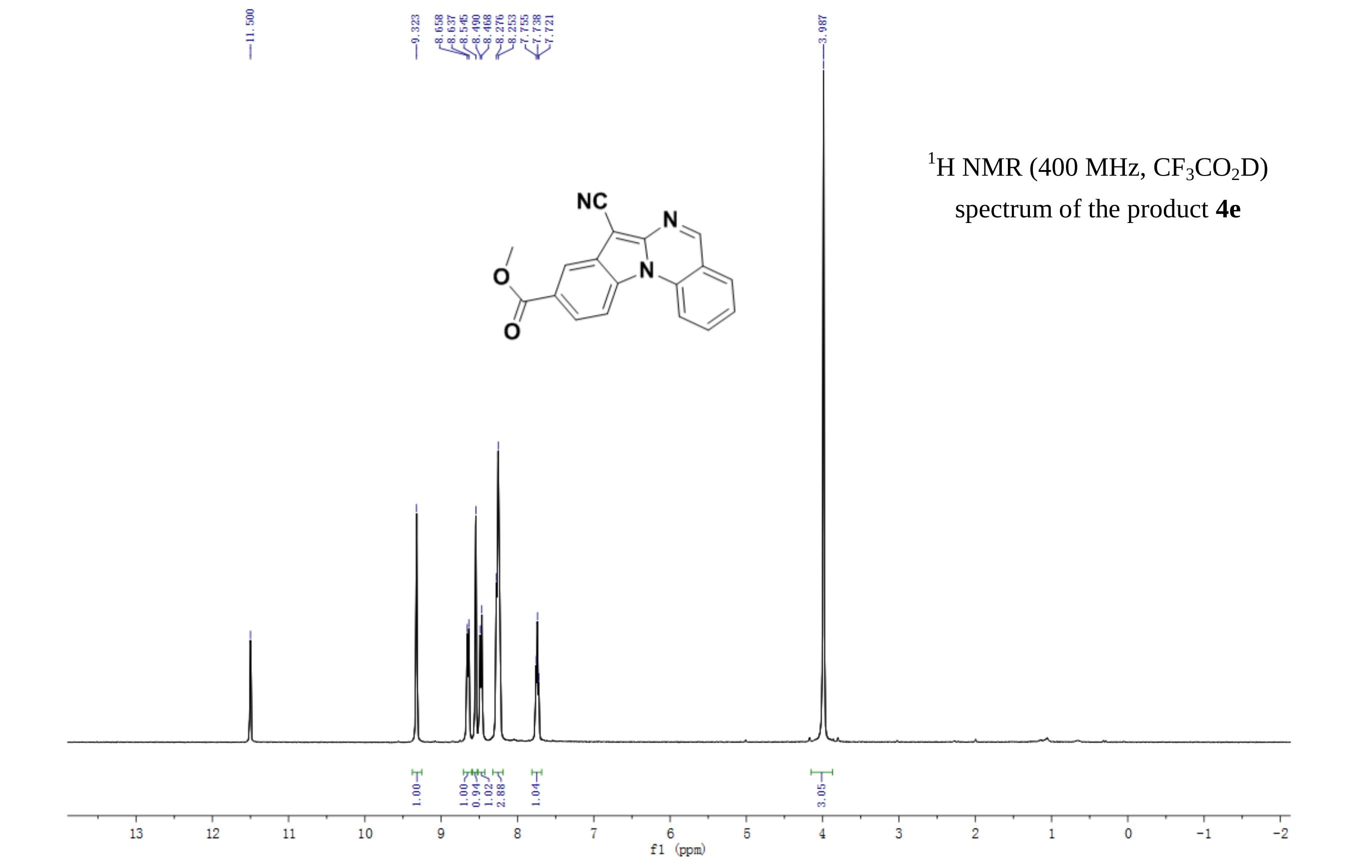

^{1}H NMR (400 MHz, CF_3CO_2D) spectrum of the product **4e**

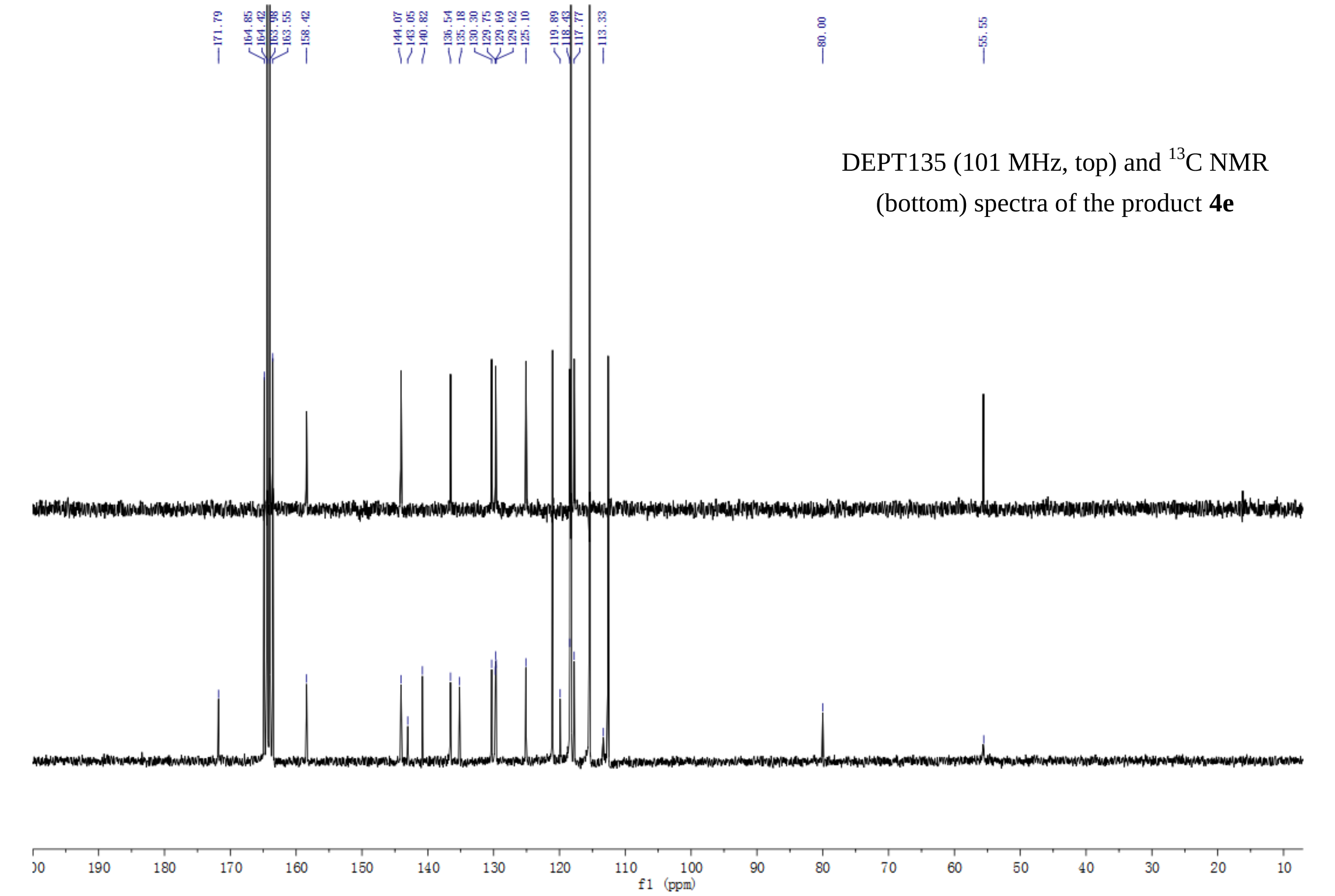

DEPT135 (101 MHz, top) and ^{13}C NMR (bottom) spectra of the product **4e**

9-Fluoroindolo[1,2-*a*]quinazoline-7-carbonitrile (4f)

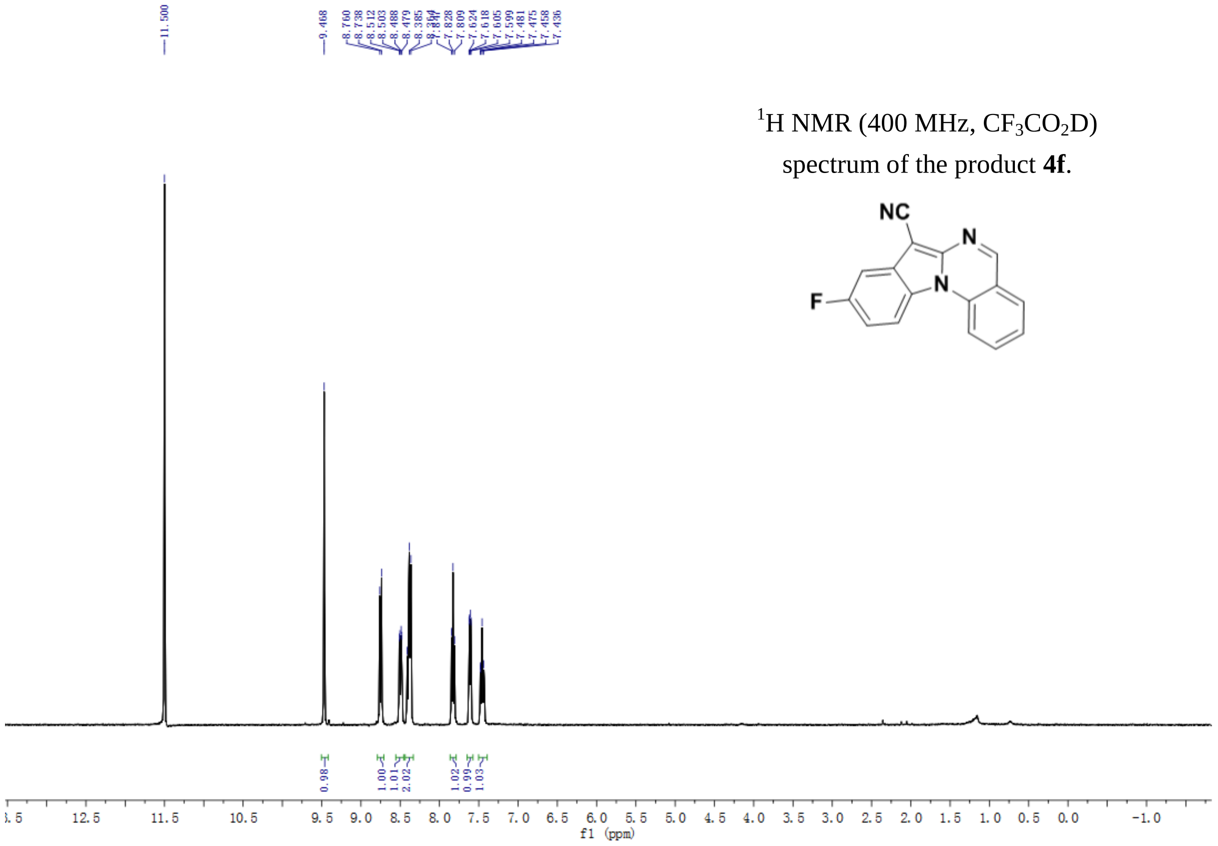

^{1}H NMR (400 MHz, CF_3CO_2D) spectrum of the product **4f**.

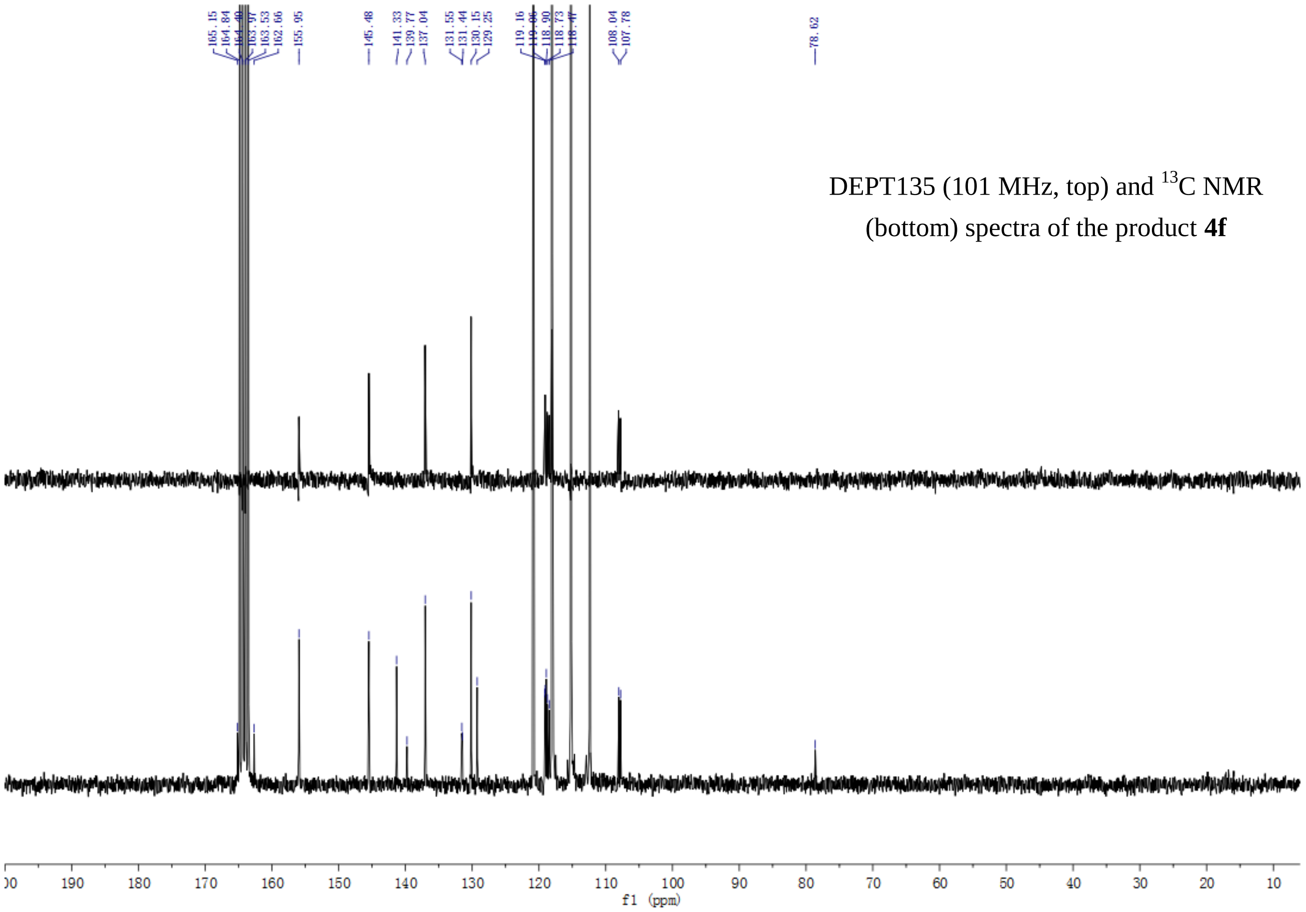

DEPT135 (101 MHz, top) and ^{13}C NMR (bottom) spectra of the product **4f**

9-Chloroindolo[1,2-*a*]quinazoline-7-carbonitrile (4g)

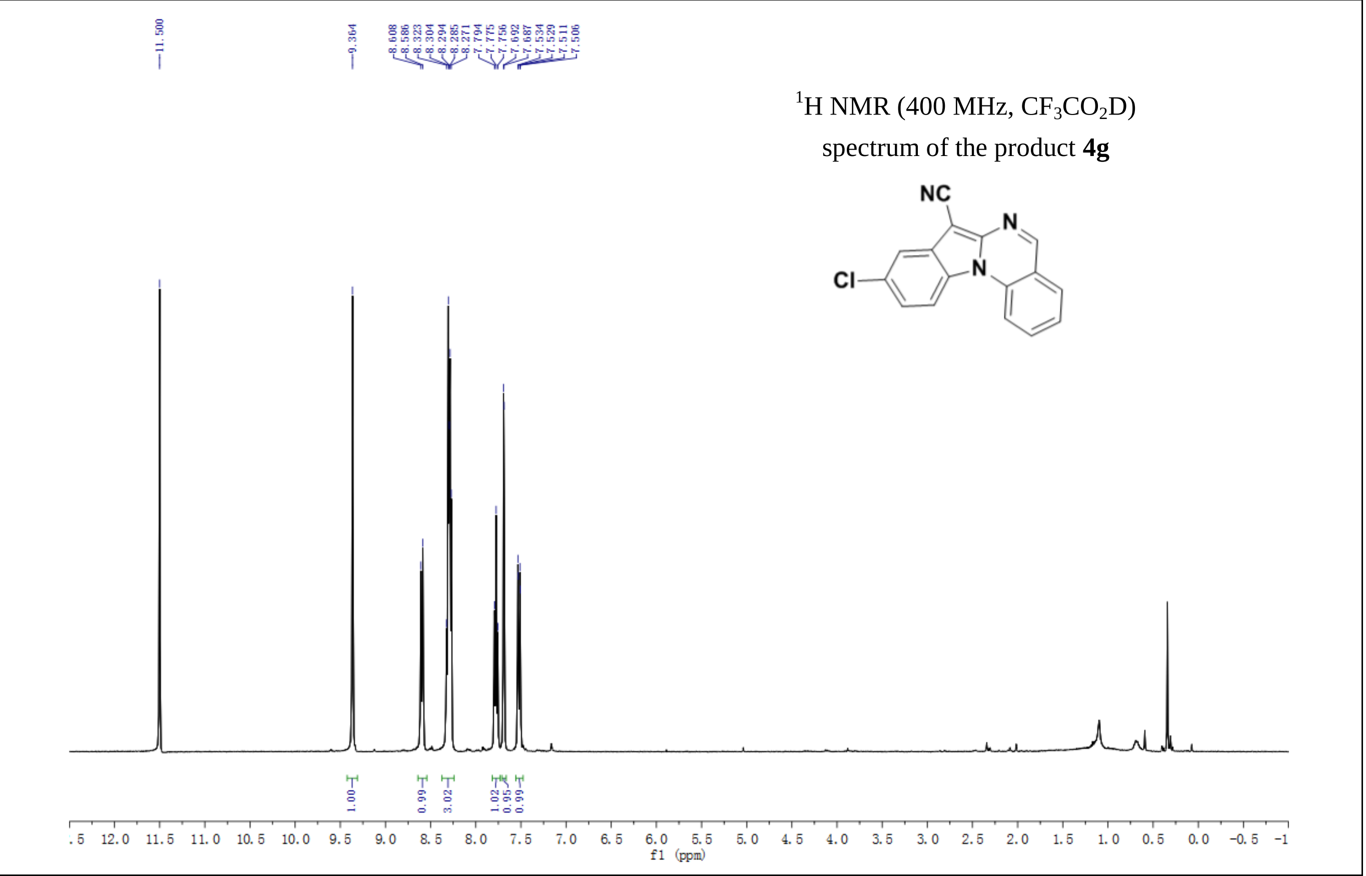

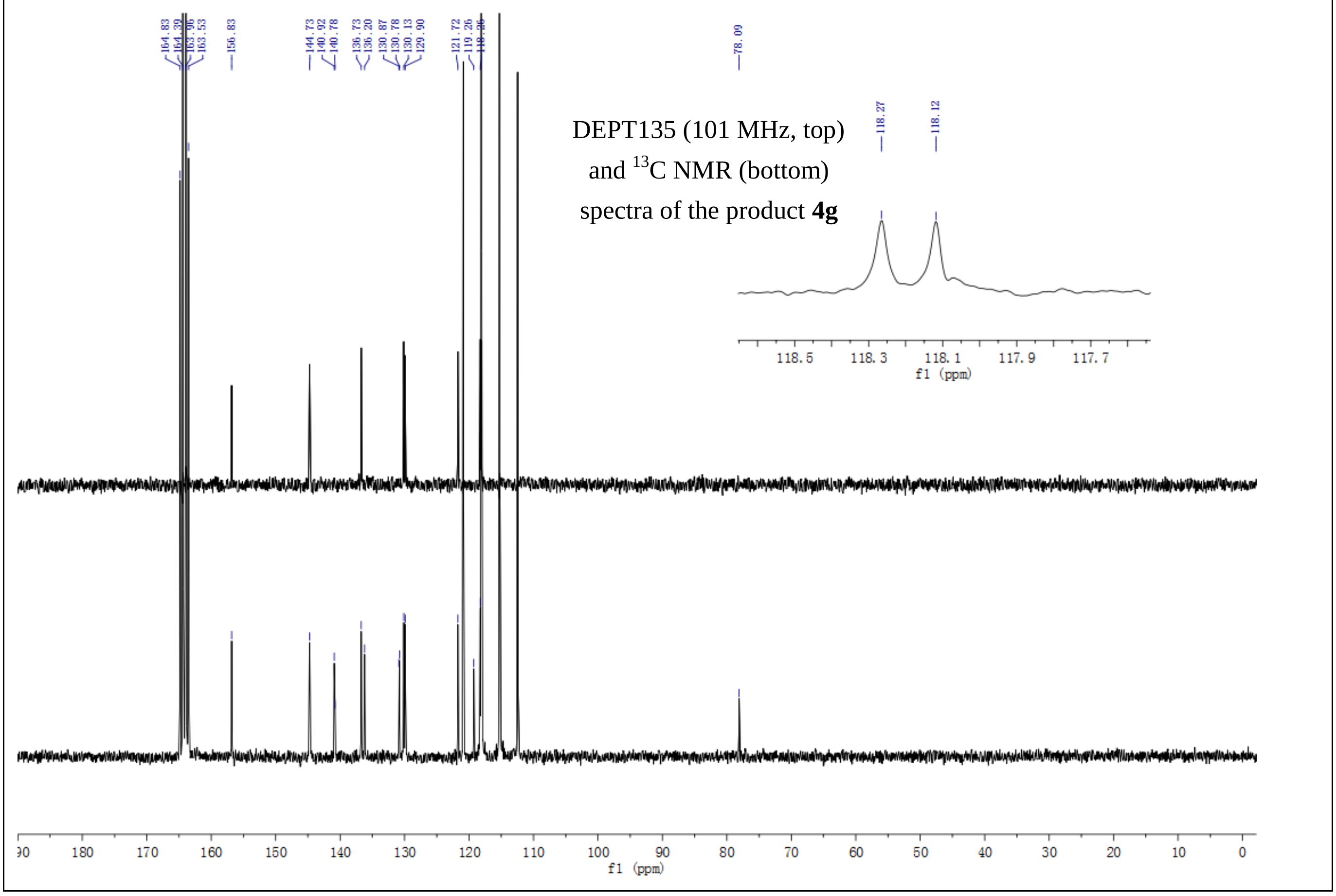

9-Bromoindolo[1,2-*a*]quinazoline-7-carbonitrile (4h)

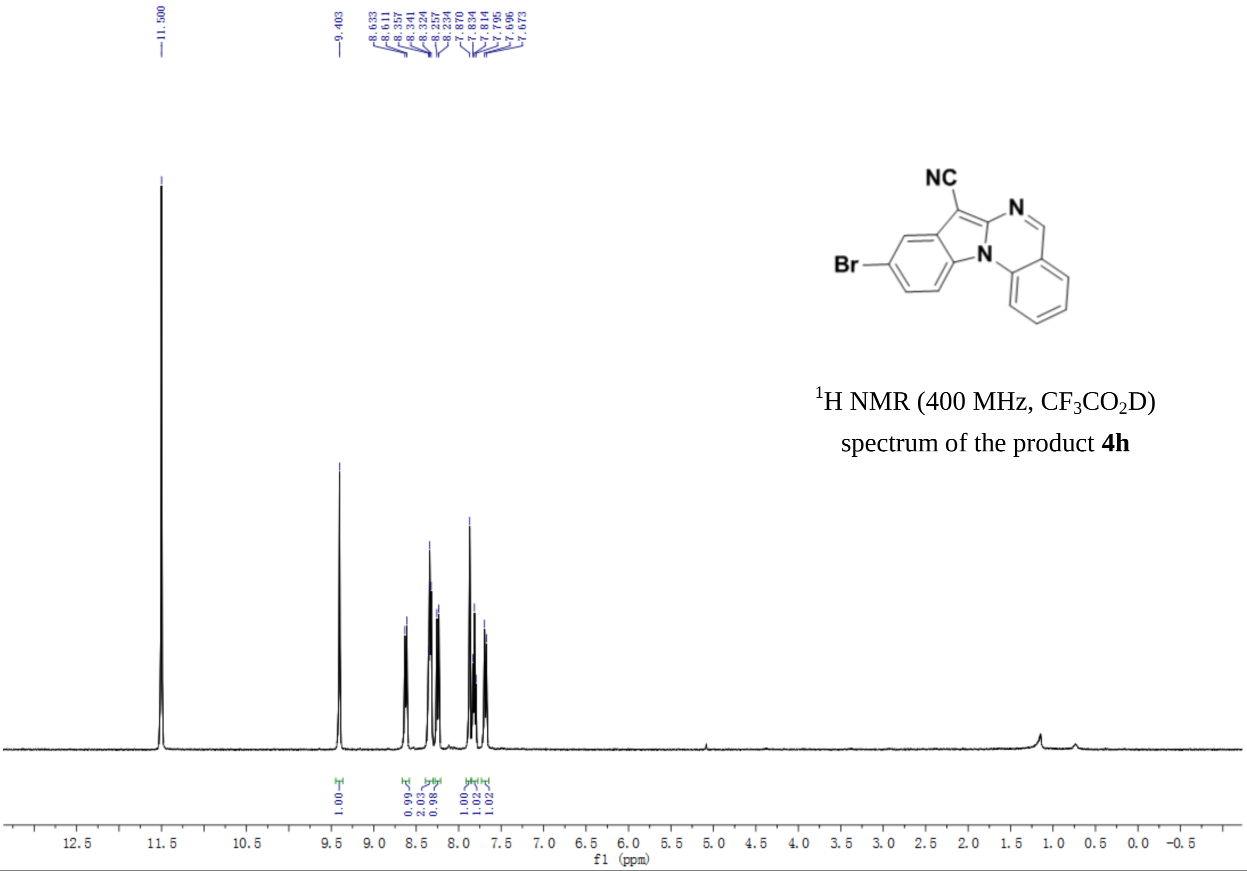

^{1}H NMR (400 MHz, CF_3CO_2D)
spectrum of the product **4h**

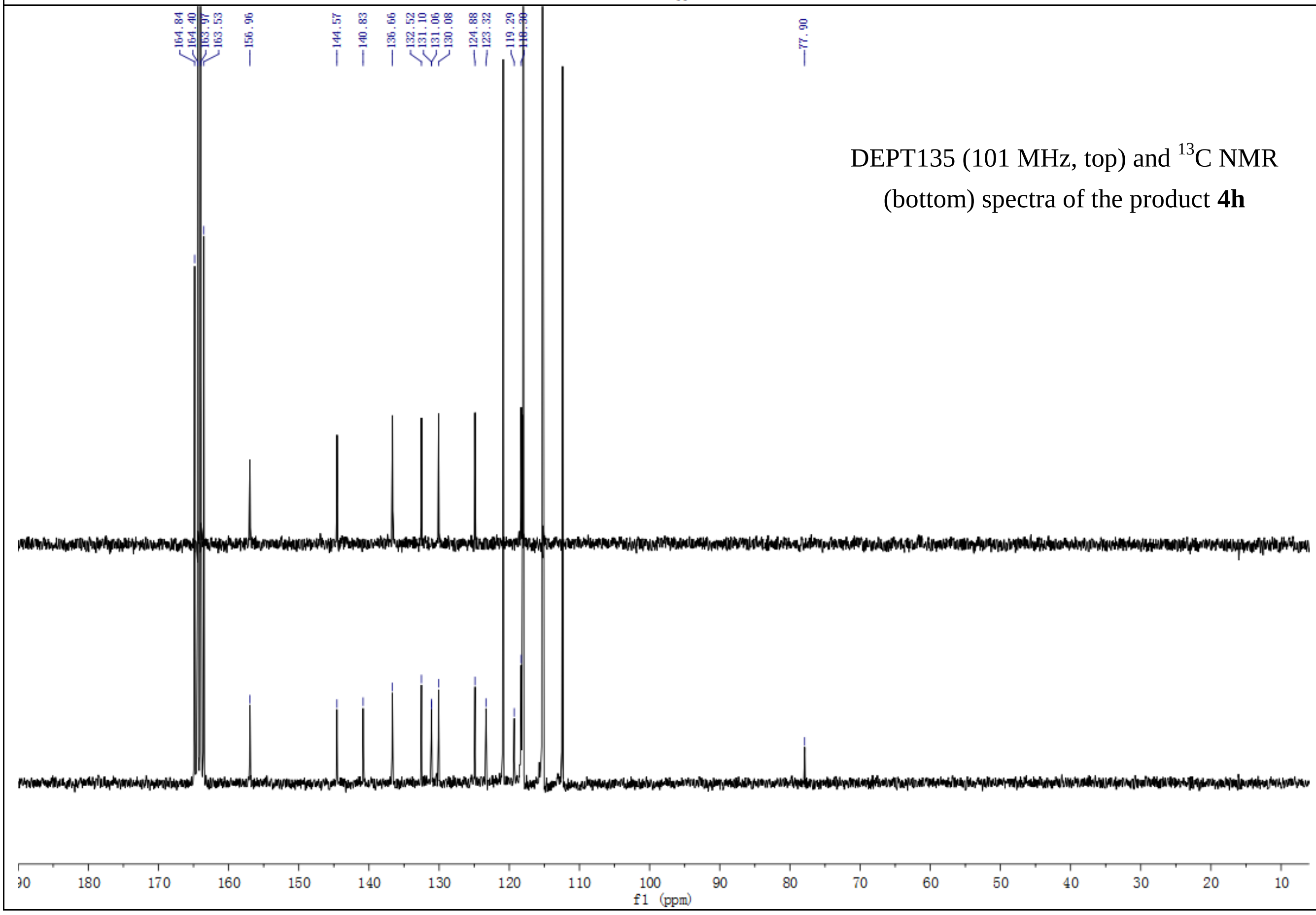

DEPT135 (101 MHz, top) and ^{13}C NMR
(bottom) spectra of the product **4h**

10-Fluoroindolo[1,2-*a*]quinazoline-7-carbonitrile (4i)

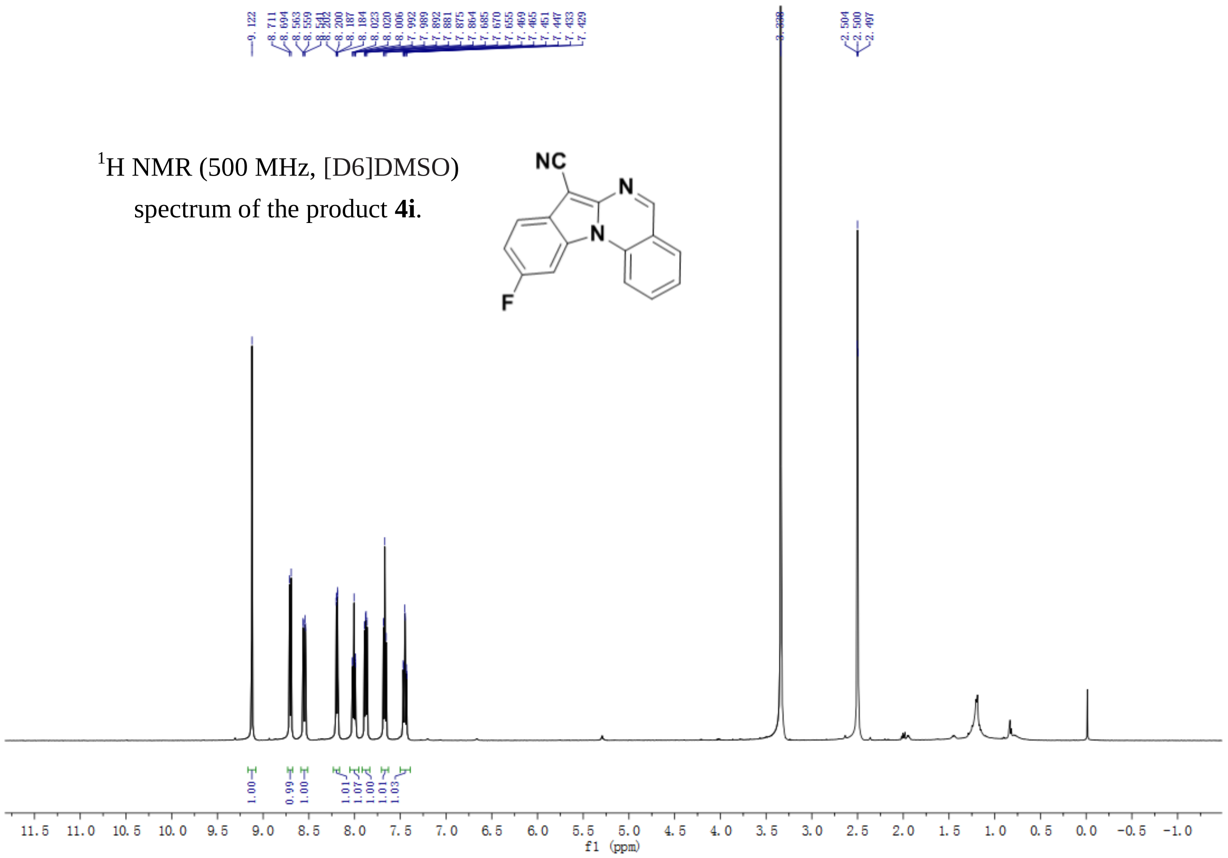

^{1}H NMR (500 MHz, [D6]DMSO) spectrum of the product **4i**.

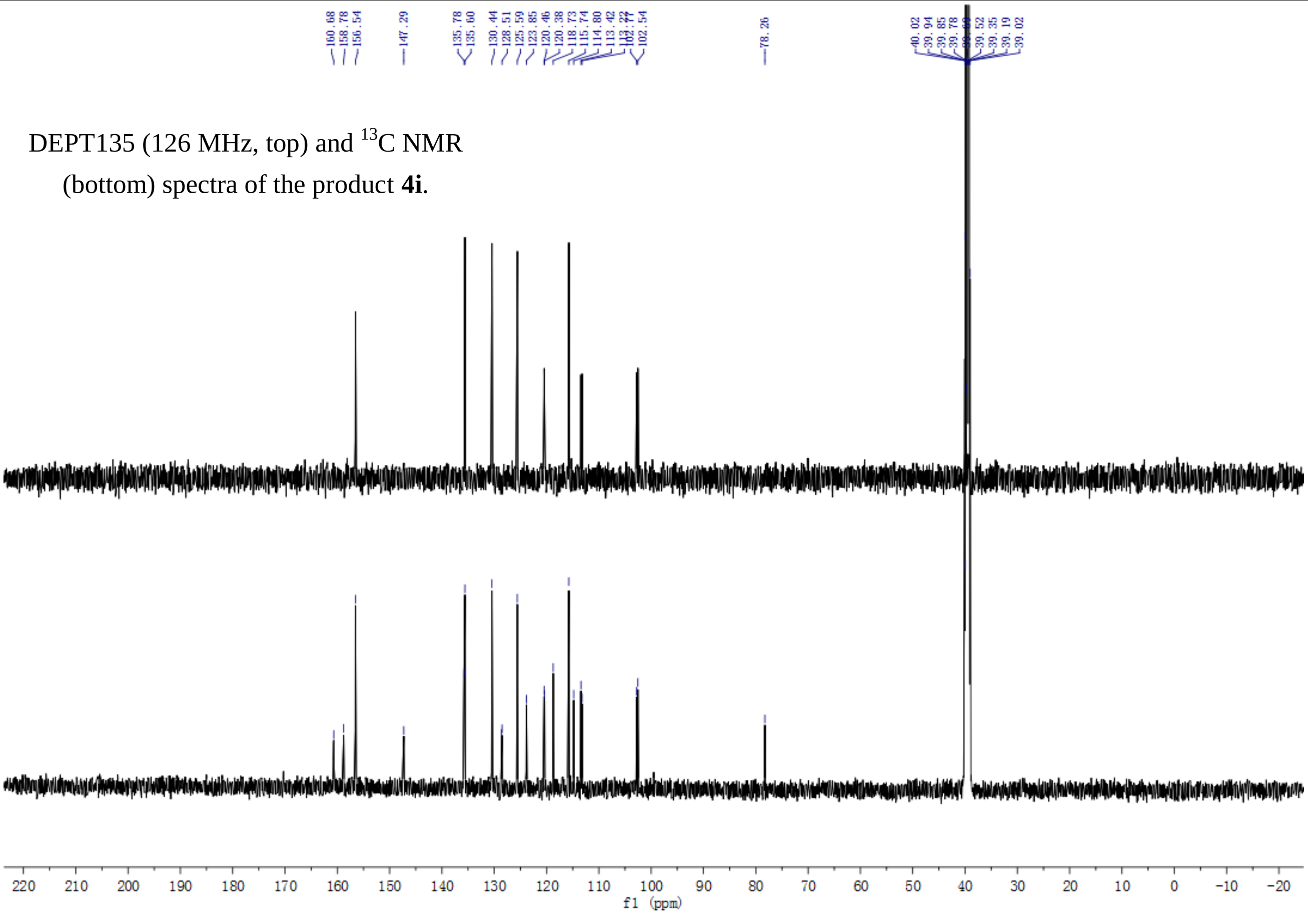

DEPT135 (126 MHz, top) and ^{13}C NMR (bottom) spectra of the product **4i**.

10-Chloroindolo[1,2-*a*]quinazoline-7-carbonitrile (4j)

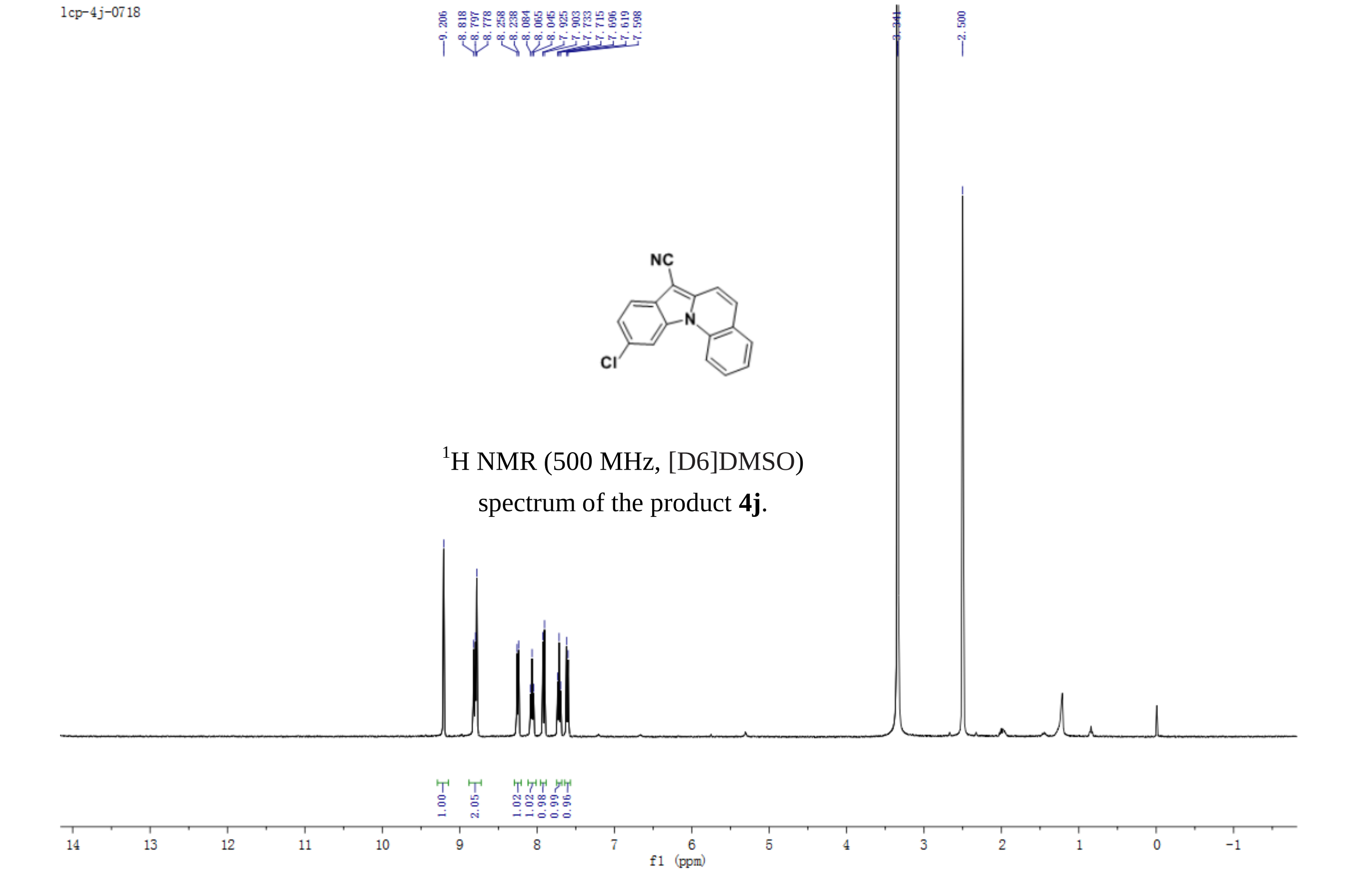

^{1}H NMR (500 MHz, [D6]DMSO) spectrum of the product **4j**.

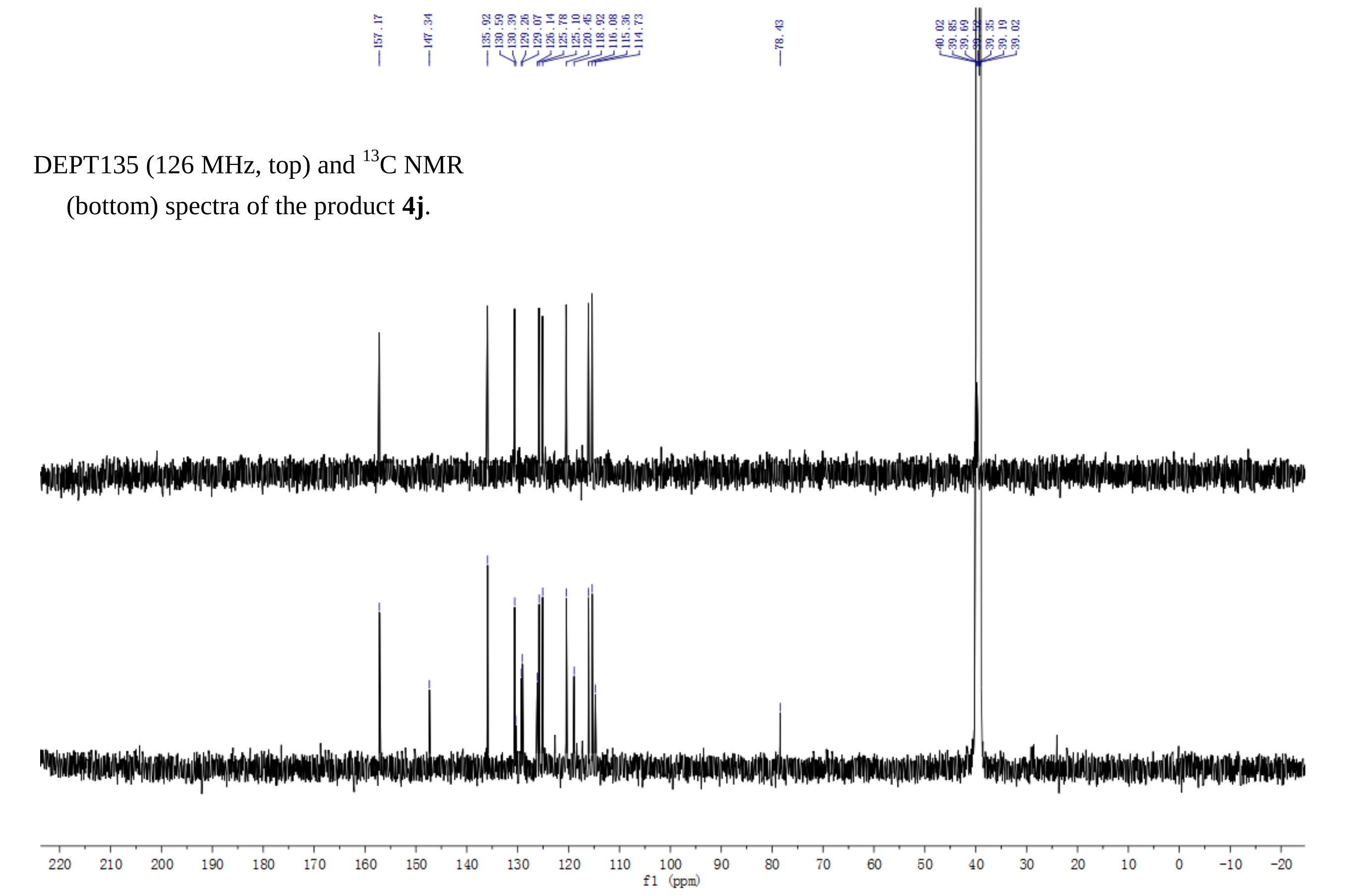

DEPT135 (126 MHz, top) and ^{13}C NMR (bottom) spectra of the product **4j**.

Methyl 7-cyanoindolo[1,2-*a*]quinazoline-10-carboxylate (4k)

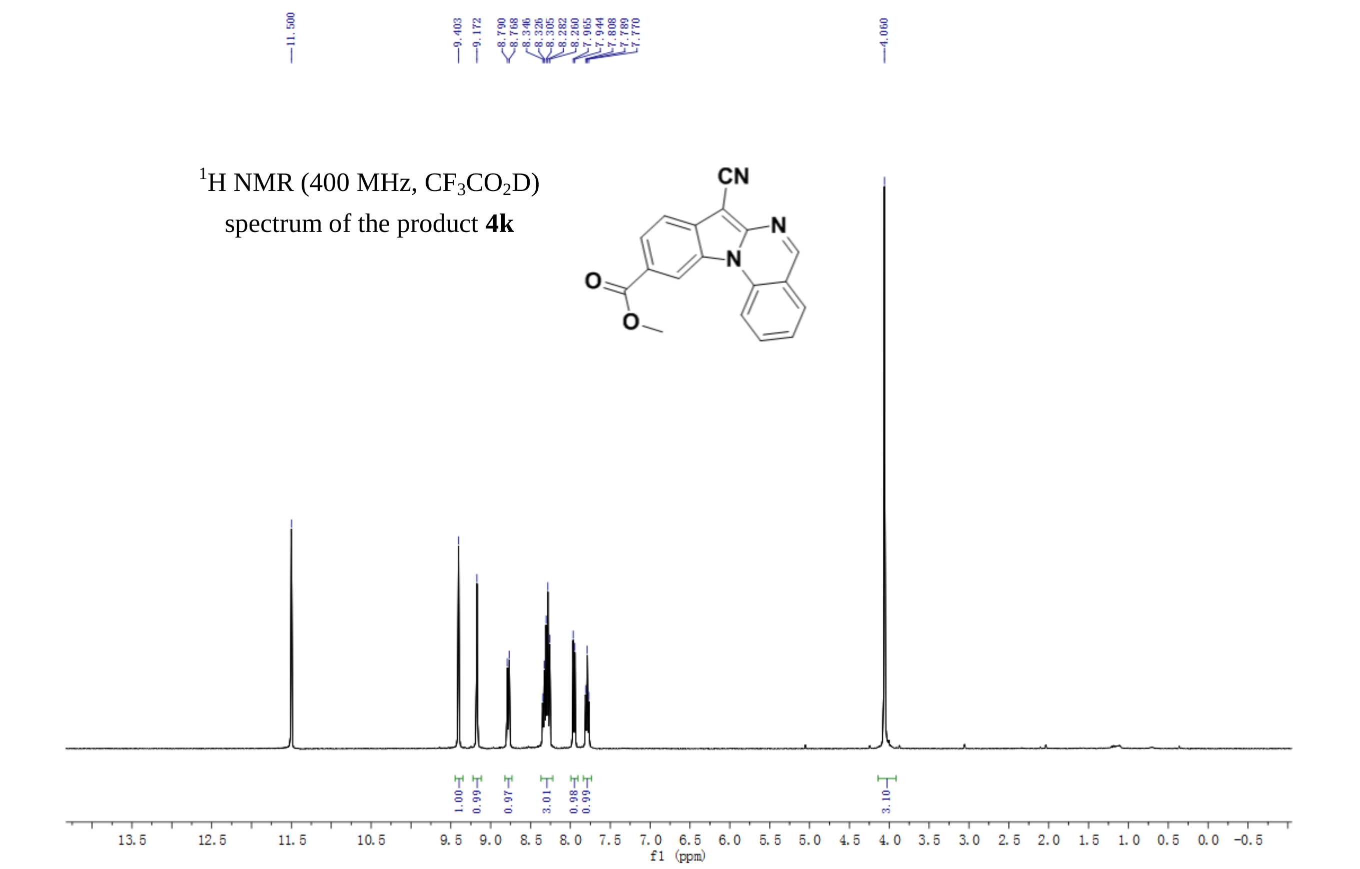

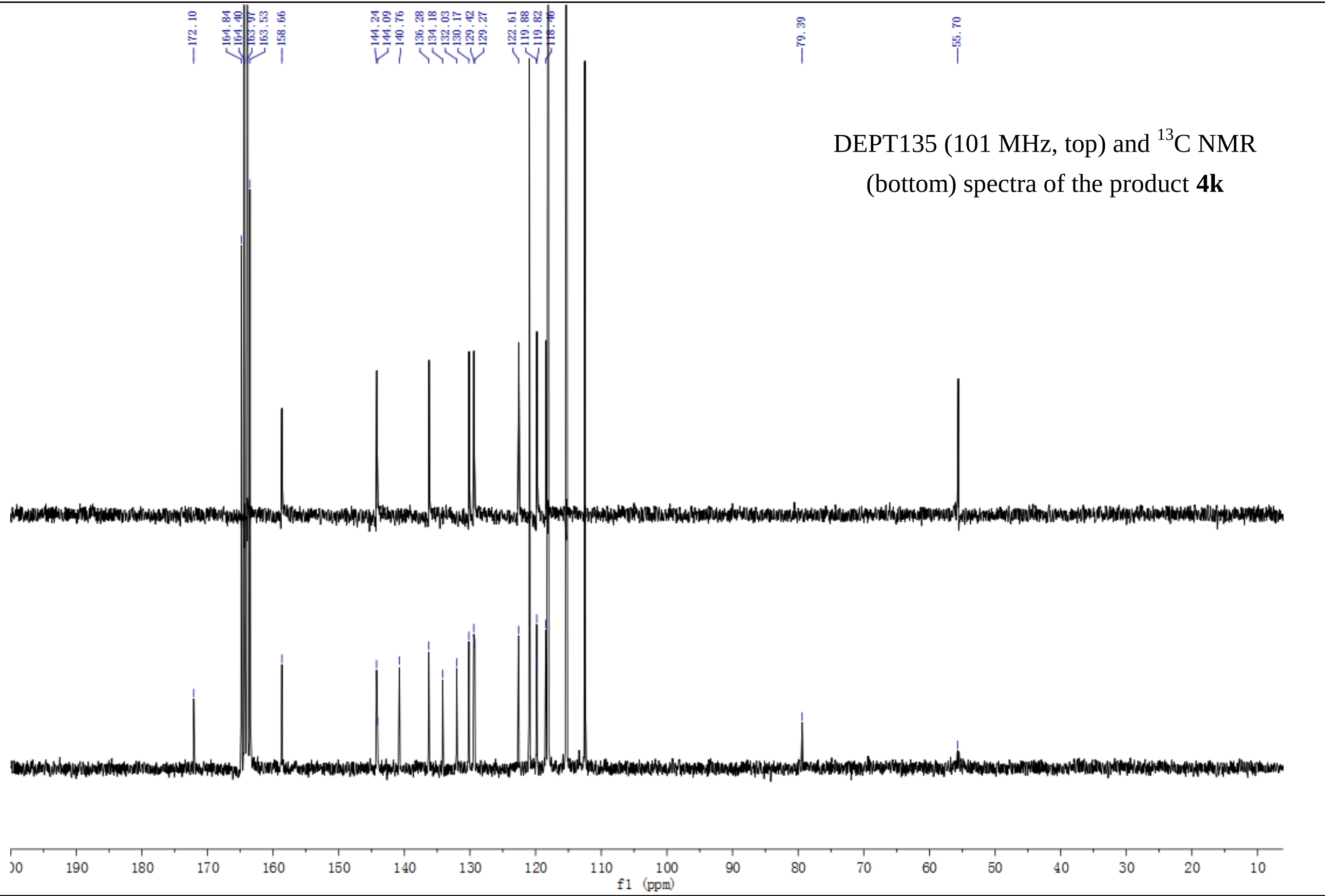

7-(Methylsulfonyl)indolo[1,2-*a*]quinazoline (4l)

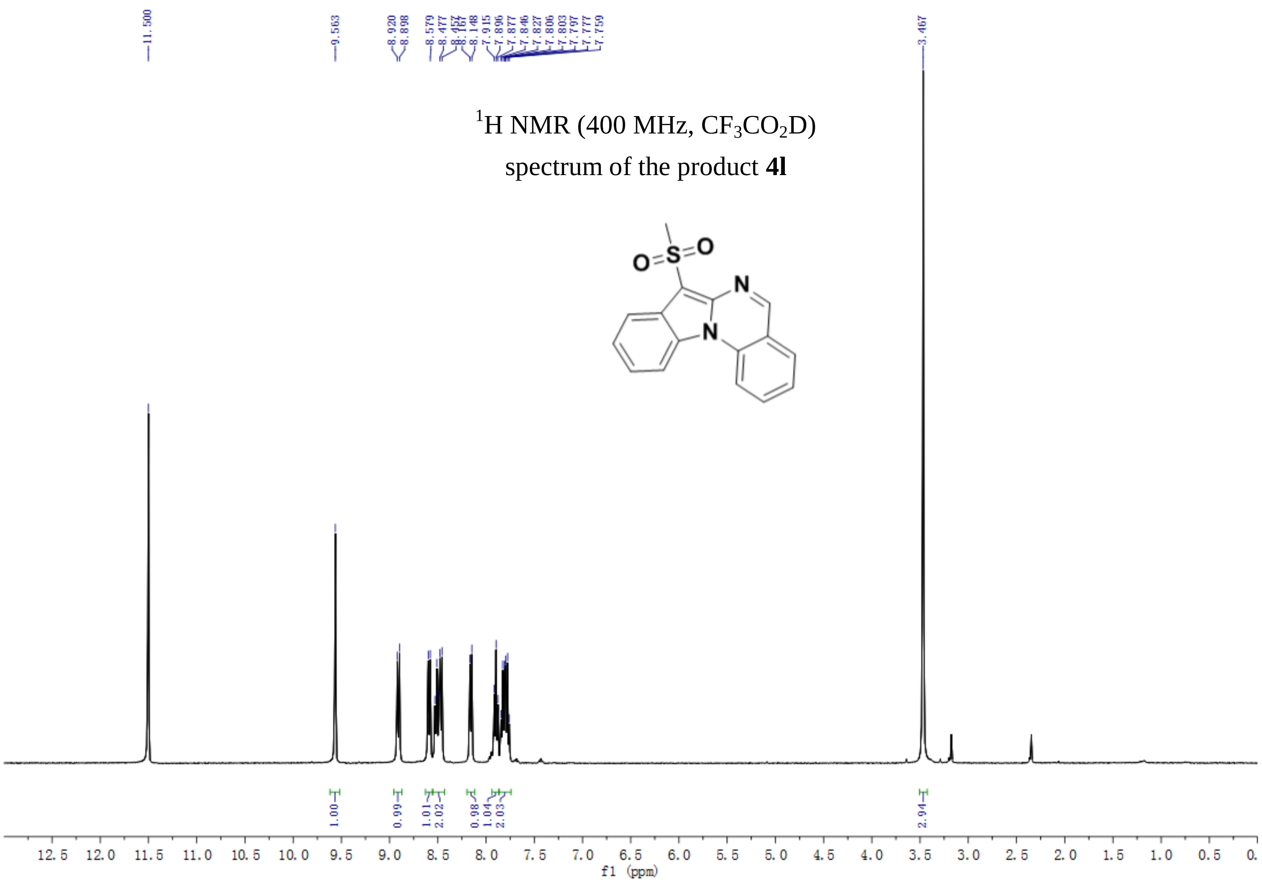

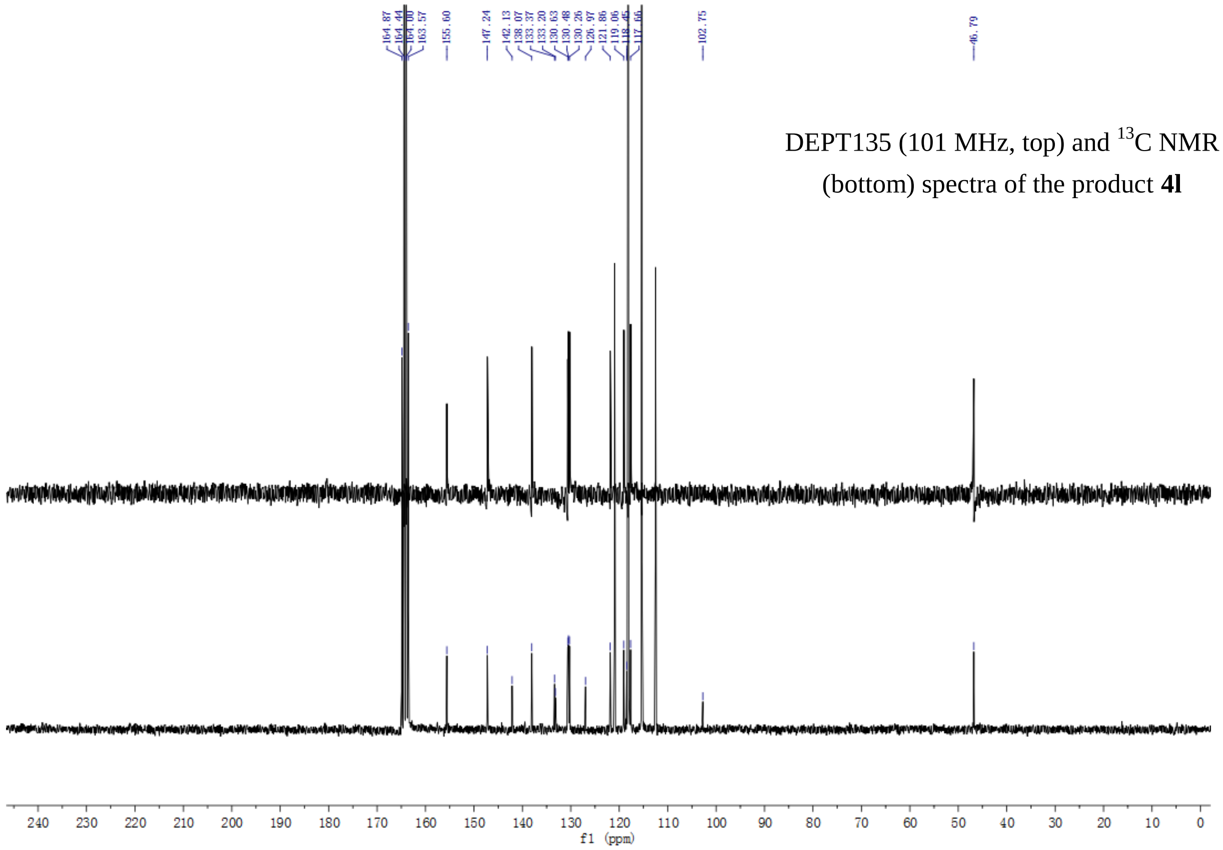

7-(Phenylsulfonyl)indolo[1,2-*a*]quinazoline (4m)

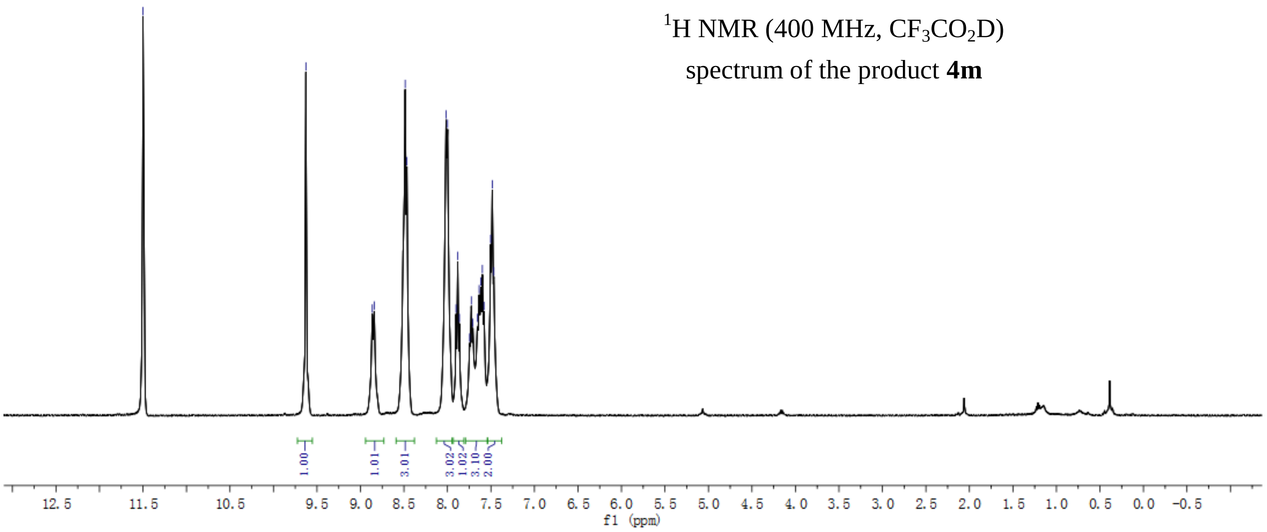

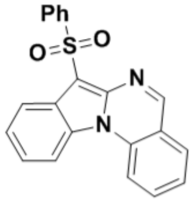

^{1}H NMR (400 MHz, CF_3CO_2D) spectrum of the product **4m**

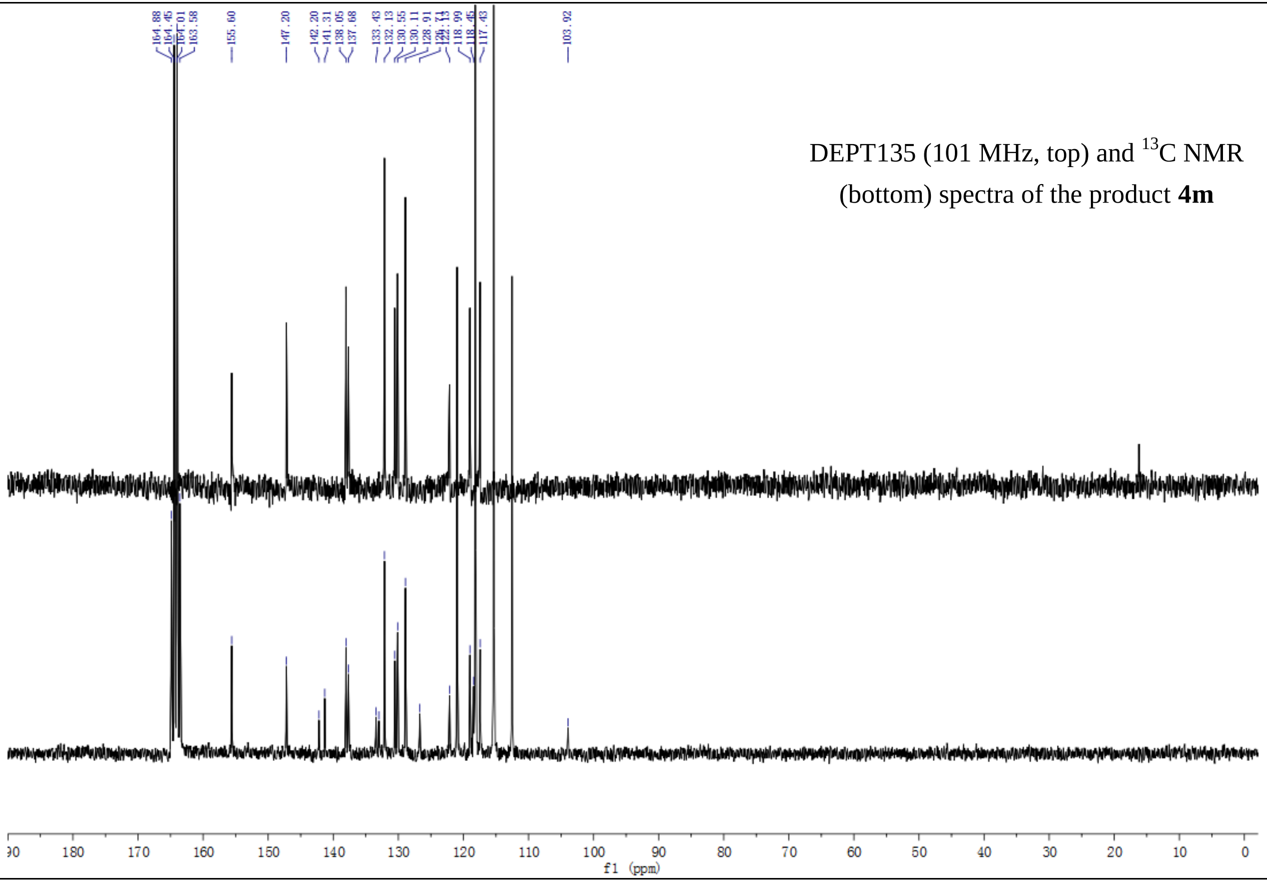

DEPT135 (101 MHz, top) and ^{13}C NMR (bottom) spectra of the product **4m**

2,3-Dimethoxyindolo[1,2-*a*]quinazoline-7-carbonitrile (4n)

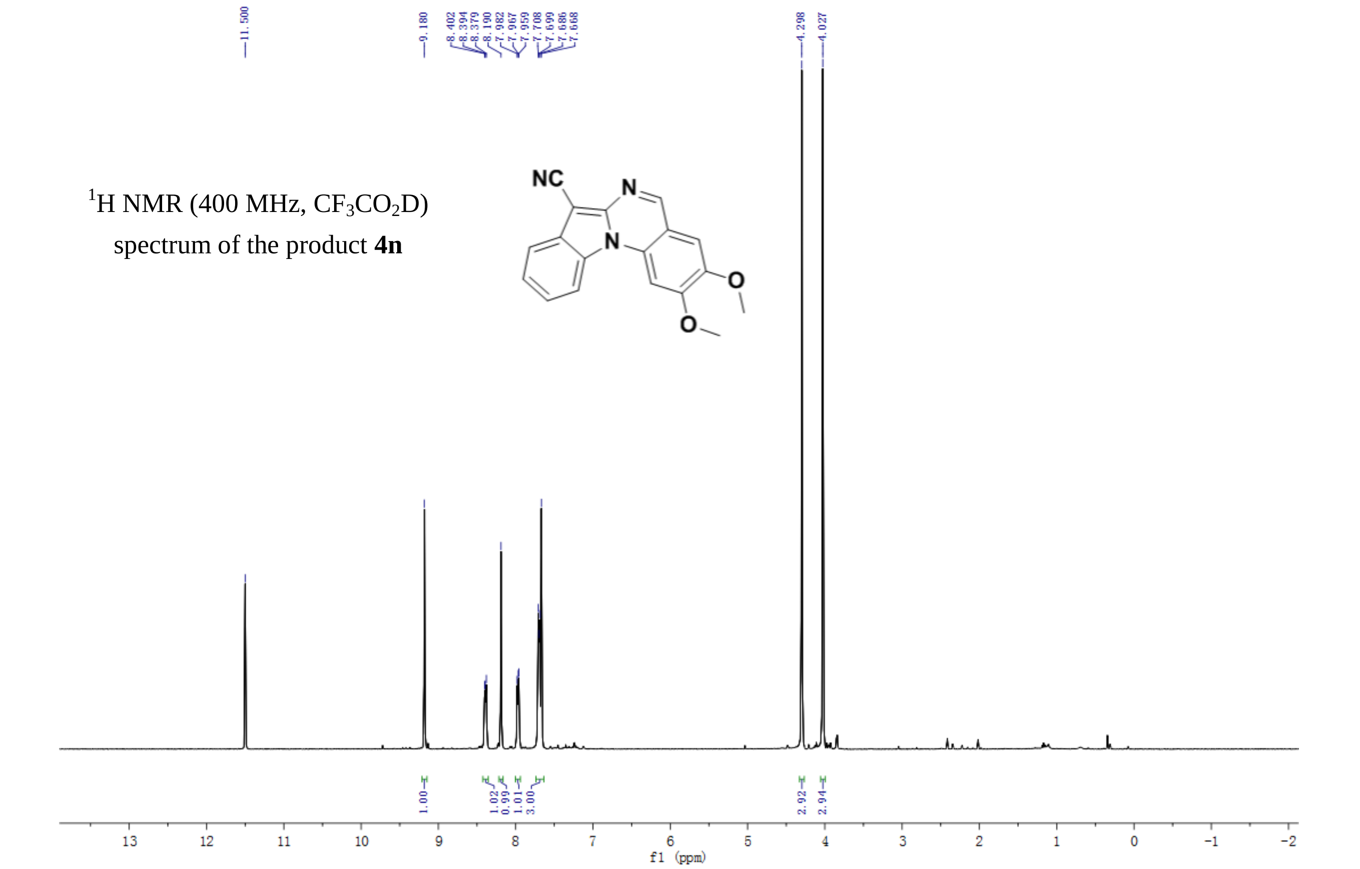

^{1}H NMR (400 MHz, CF_3CO_2D) spectrum of the product **4n**

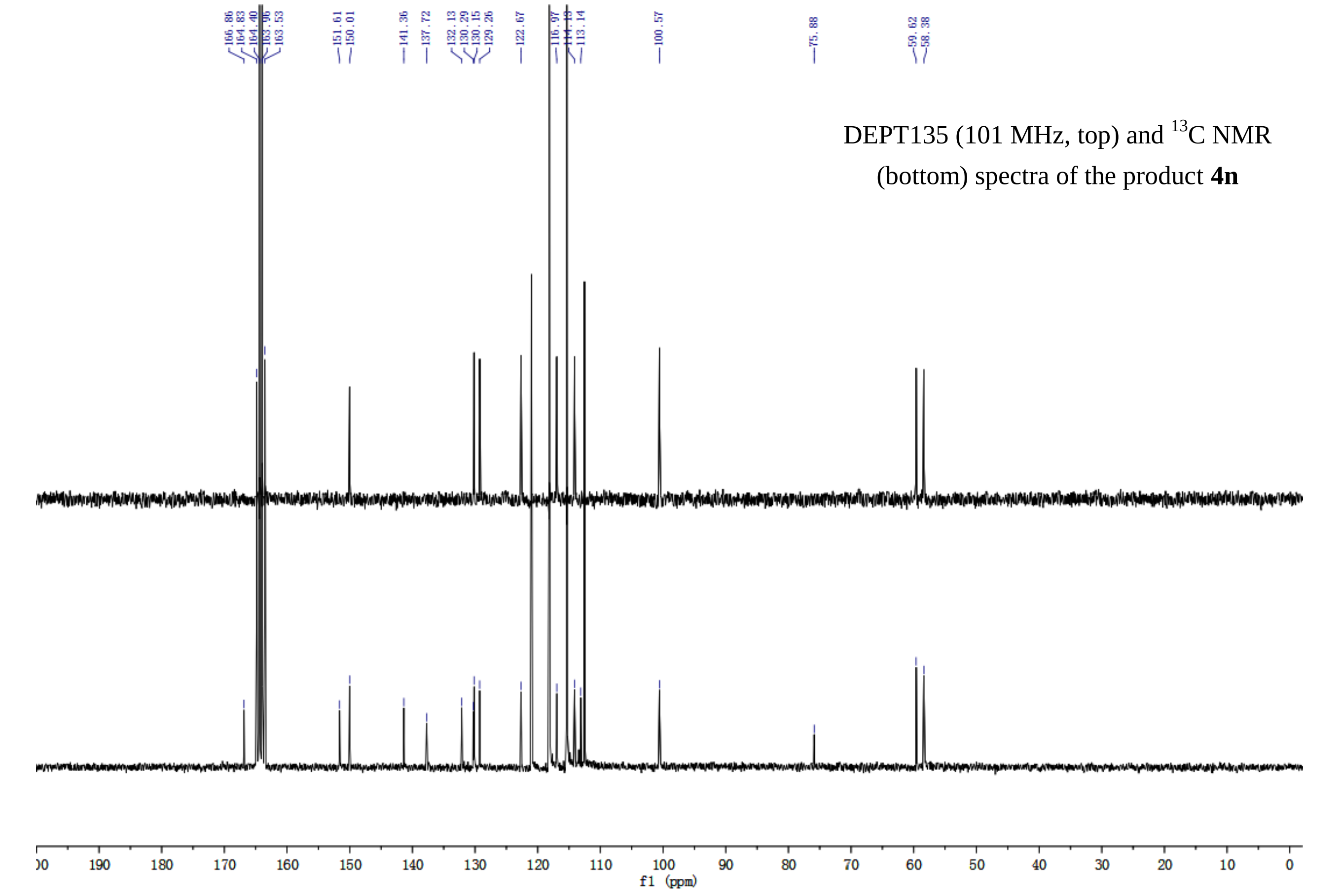

DEPT135 (101 MHz, top) and ^{13}C NMR (bottom) spectra of the product **4n**

3-Methylindolo[1,2-*a*]quinazoline-7-carbonitrile (4o)

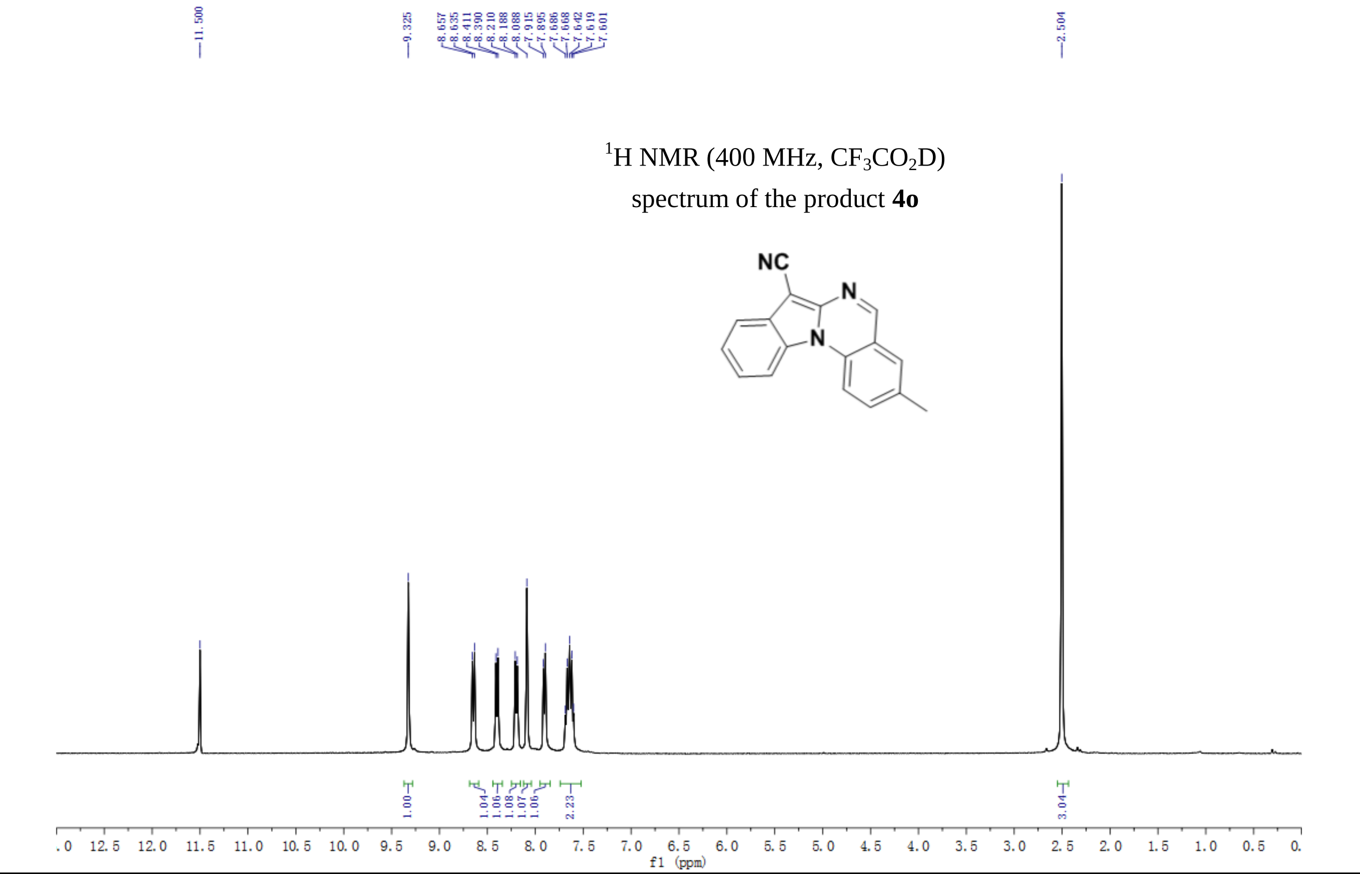

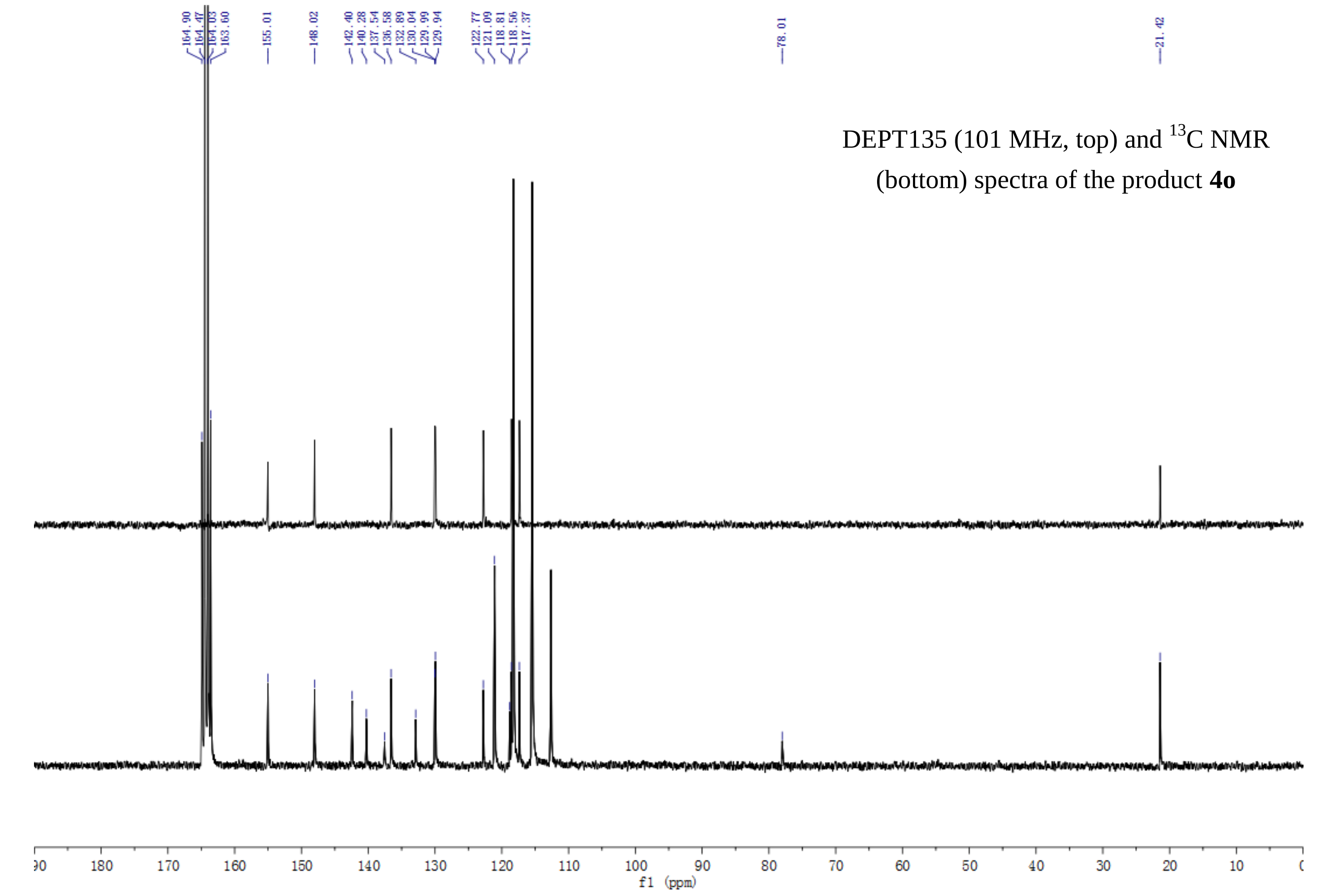

2-Fluoroindolo[1,2-*a*]quinazoline-7-carbonitrile (4p)

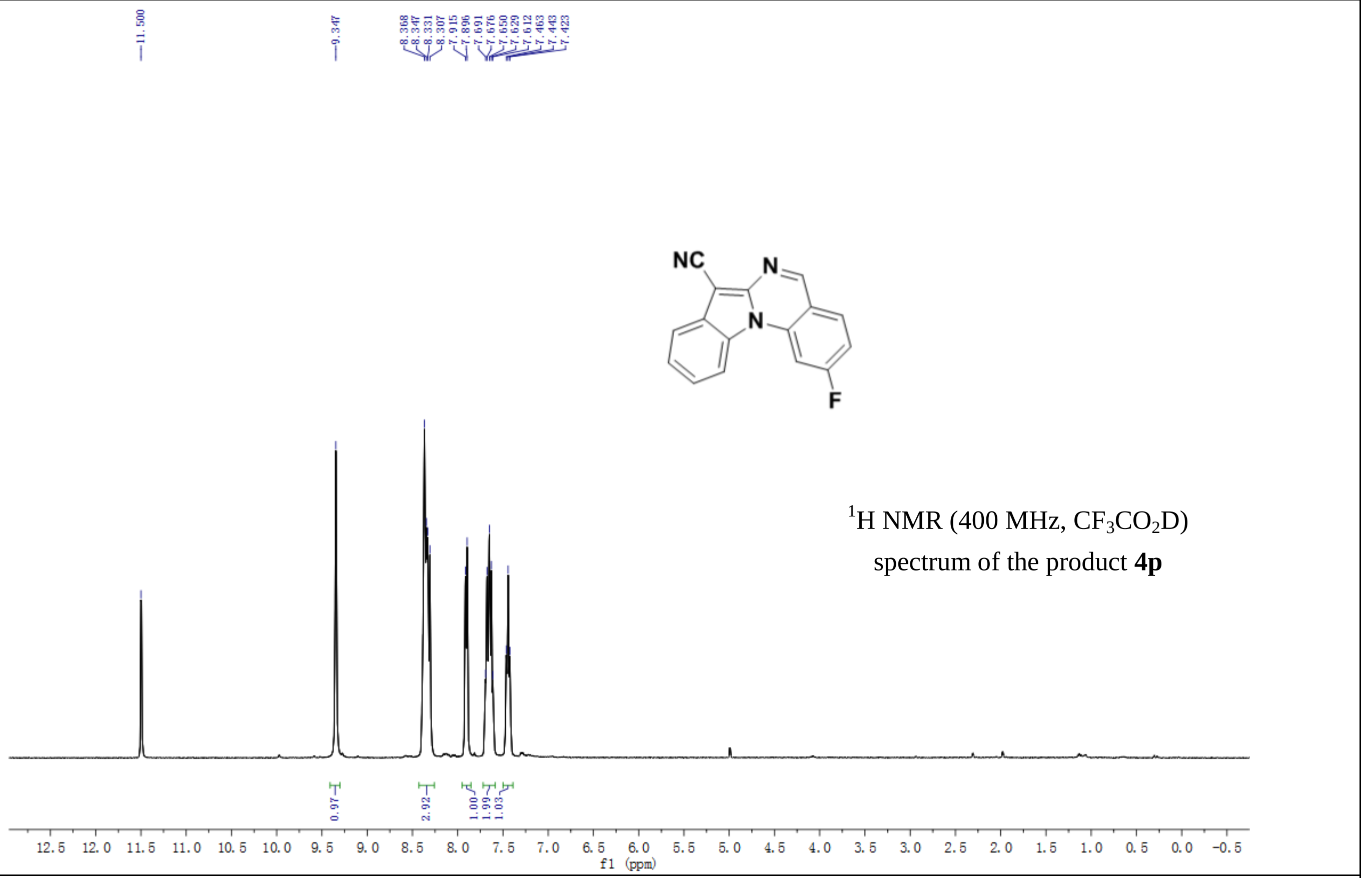

^{1}H NMR (400 MHz, CF_3CO_2D) spectrum of the product **4p**

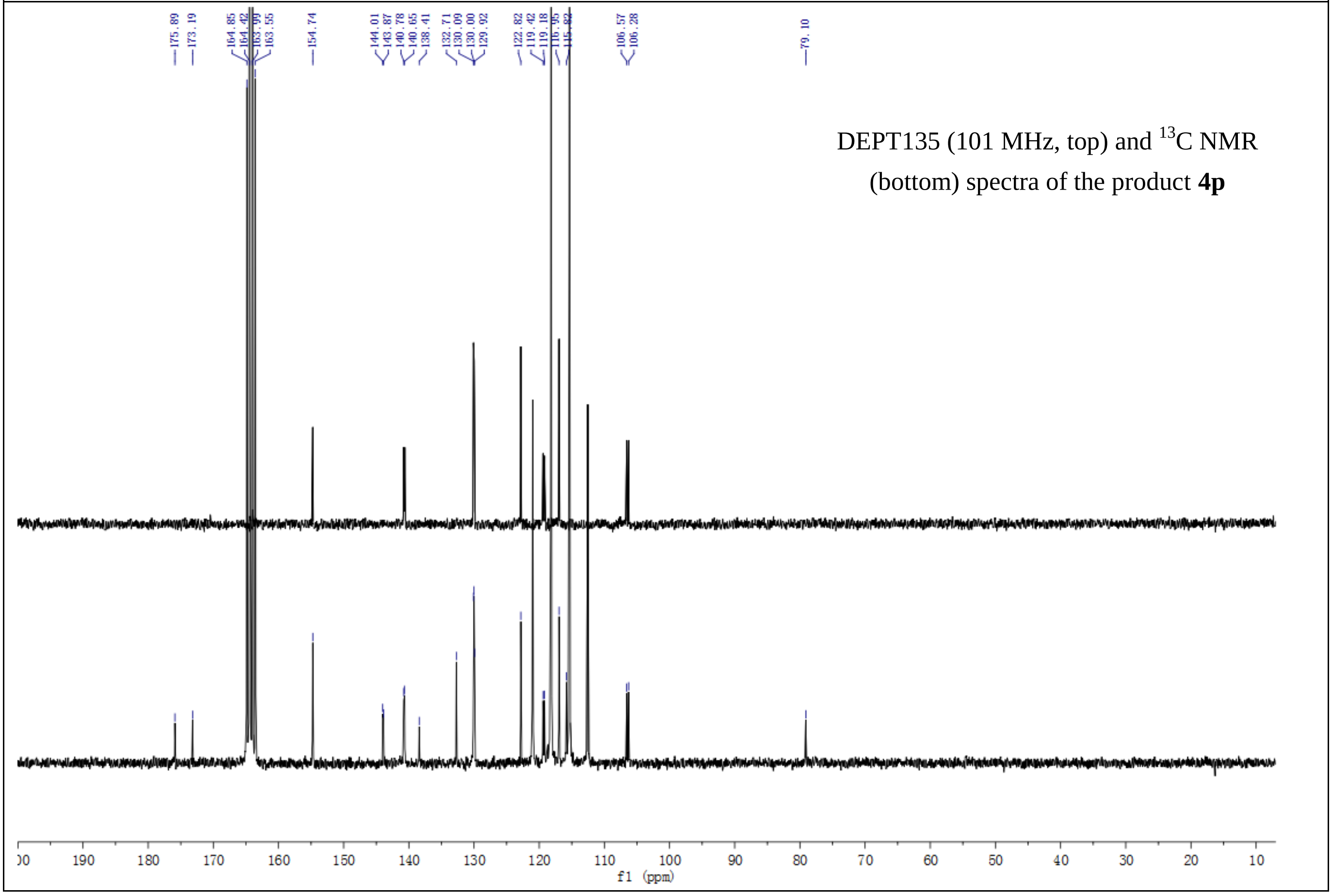

DEPT135 (101 MHz, top) and ^{13}C NMR (bottom) spectra of the product **4p**

2-Chloroindolo[1,2-*a*]quinazoline-7-carbonitrile (4q)

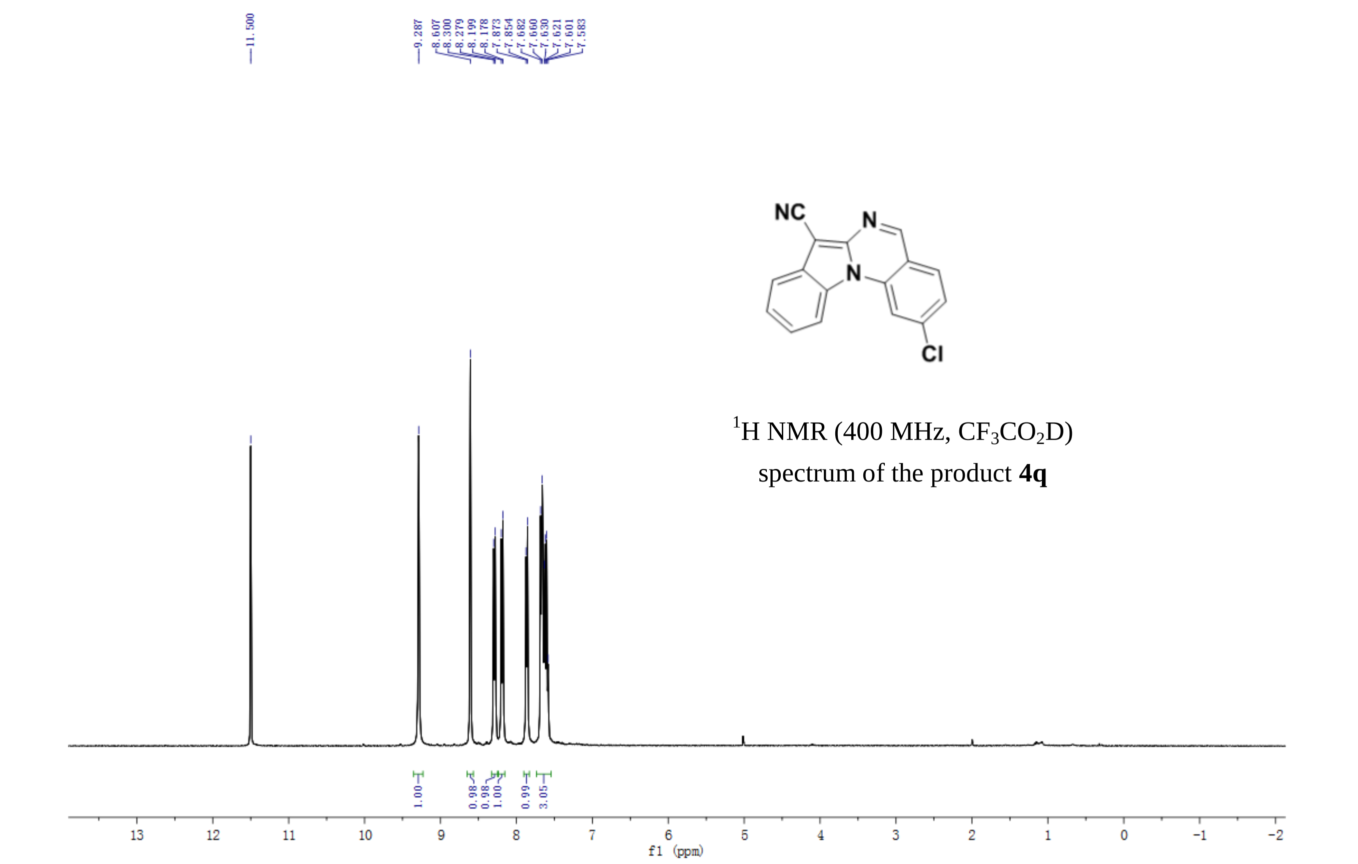

^{1}H NMR (400 MHz, CF_3CO_2D) spectrum of the product **4q**

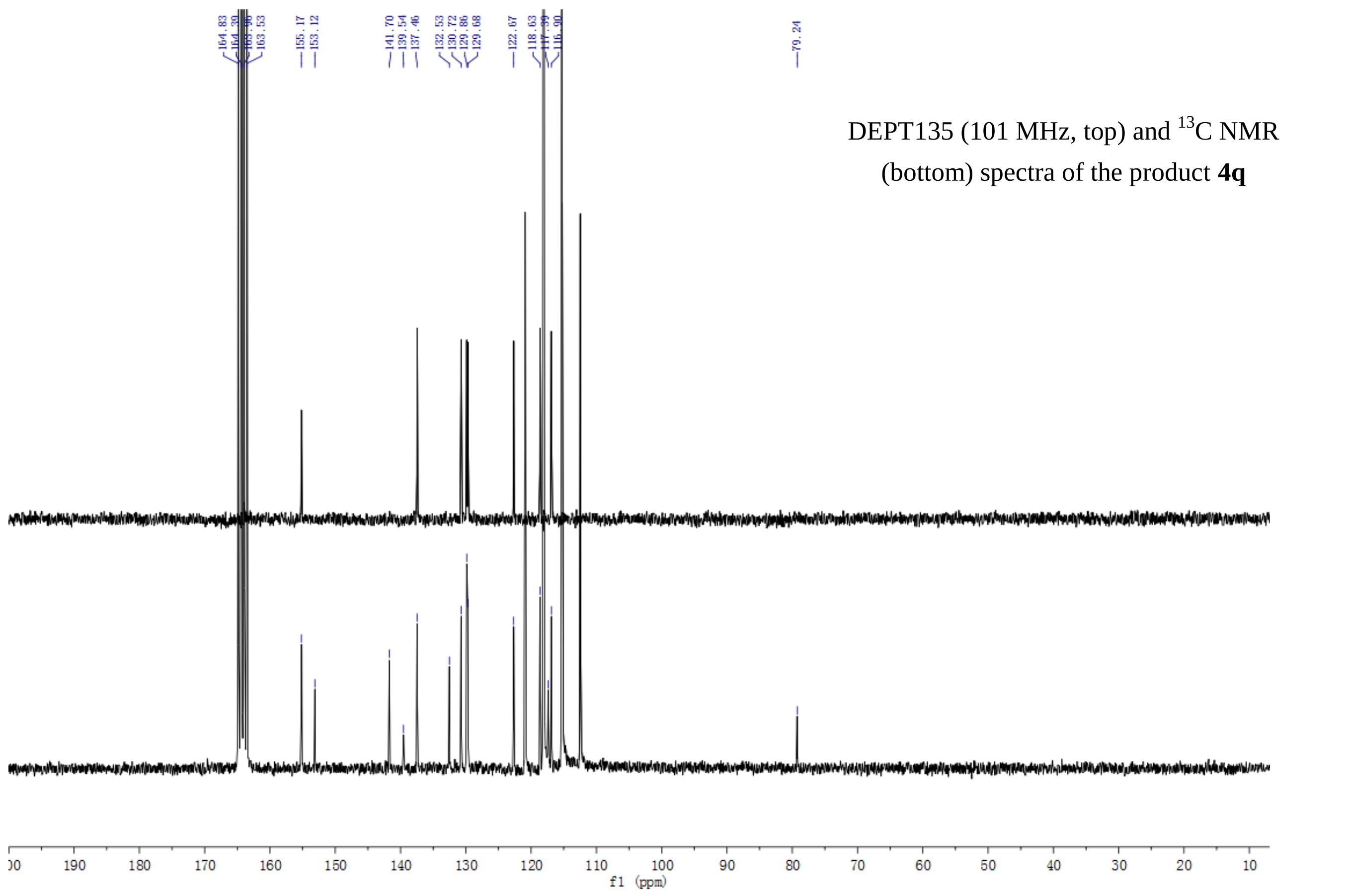

DEPT135 (101 MHz, top) and ^{13}C NMR (bottom) spectra of the product **4q**
